# Few-femtosecond photoelectron spectroscopy reveals fundamental photodynamics of the carbon-carbon double bond in ethylene

**DOI:** 10.1038/s41467-026-75782-3

**Published:** 2026-07-23

**Authors:** Arnab Sen, Simon P. Neville, Martin Kretschmar, Martha Yaghoubi Jouybari, Rostyslav Danylo, José R. C. Andrade, Marc J. J. Vrakking, Albert Stolow, Tamas Nagy, Michael S. Schuurman, Arnaud Rouzée

**Affiliations:** 1 Max-Born-Institut, Berlin, Germany; 2 National Research Council Canada, Ottawa, ON Canada; 3 Department of Chemistry and Biomolecular Sciences, University of Ottawa, Ottawa, ON Canada; 4 Department of Physics, University of Ottawa, Ottawa, ON Canada; 5 NRC-uOttawa Joint Centre for Extreme Photonics, University of Ottawa, Ottawa, ON Canada

**Keywords:** Photochemistry, Atomic and molecular physics, Excited states

## Abstract

Conjugated *π*-systems, often composed of carbon-carbon double bonds as key structural units, are among the most important organic chromophores and serve as prototypical systems for studying ππ^*^ excitation. Their ultrafast excited-state dynamics govern the outcomes of fundamental photochemical processes, including vision, photoisomerization, photosynthesis, and solar energy conversion. Here, we investigate the ultrafast dynamics of the simplest isolated C=C chromophore, ethylene, and its per-deuterated isotopologue using broadly tunable sub-4 femtosecond pulses in the 150-200 nm range combined with time-resolved photoelectron spectroscopy. Supported by advanced quantum dynamics calculations, our results reveal the important role of the ^1^*B*_3*g*_(σπ^*^) state, which is strongly coupled through torsional motion to the optically populated ^1^*B*_1*u*_(ππ^*^) state. These findings establish a revised framework for understanding and controlling photochemical reaction in conjugated molecular systems.

## Abstract (web summary)

The authors show that ultrafast nonadiabatic coupling transfers population from the optically excited ππ^*^ state to the σπ^*^ state in ethylene within 10 fs using sub-4 fs time-resolved photoelectron spectroscopy and quantum dynamics simulations.

## Introduction

The absorption of light by chromophores governs some of the most important processes on Earth^1,2^, including photosynthesis^3,4^, vision^5,6^, and photobiochemistry^7^, as well as technologically important photovoltaic and other solar energy conversion processes^8,9^. A fundamentally important organic chromophore is the carbon-carbon double bond, as its dynamics upon absorption of light can lead to cis-trans isomerization, often the first step in light-energy conversion, molecular switches, biochemical signaling, and photochemistry. Ethylene (ethene) is the smallest organic molecule with a carbon-carbon double bond and has long served as the canonical paradigm for cis-trans isomerization^10^. Its ultrafast dynamics involve the non-adiabatic coupling of electronic and vibrational degrees of freedom—a failure of the Born-Oppenheimer approximation—which underlies our basic concepts of molecular structure and much of spectroscopy. These interactions also play a central role in the ultrafast electronic relaxation of the C${}_{2}{{{{\rm{H}}}}}_{4}^{+}$ cation, as recently observed^11,12^. Such rapid and complex couplings govern the fate of photoexcited molecules^13^, particularly the branching between different chemical outcomes.

Despite its fundamental importance, the complexity and short time scales of excited state dynamics in ethylene have to date prevented a full understanding of its primary response to the absorption of light. The absorption spectrum of ethylene in the photon energy of 6-8 eV contains two prominent features, traditionally assigned as a *π*3*s* transition to a Rydberg ^1^*B*_2*u*_ state and a ππ^*^ transition to a valence ^1^*B*_1*u*_ state. Following ππ^*^ excitation, it is accepted that, within the first 50 fs, large-amplitude torsional motion and pyramidalization about the carbon-carbon double bond lead to a twisted-orthogonal structure in which the two CH_2_ groups are rotated with respect to each other by 90^∘^. This twisted molecule then electronically relaxes to the ground state through various conical intersections^14–28^, eventually followed by fragmentation leading to both H atom and H_2_ molecule photoproducts^29,30^. Recent studies have also discussed the possible influence of the *π*3s Rydberg state on the excited state dynamics^18,25^, as well as of additional complex dynamics leading to the possible fragmentation of the molecule occurring in less than 20 fs^26^.

Here we present the highest time resolution study to date of the ultrafast excited state dynamics of ethylene, using broadly tunable sub-4 fs Vacuum UltraViolet (VUV) pump and probe laser pulses^31^ combined with time-resolved photoelectron spectroscopy (TRPES) detection^32–37^. In parallel, we employ state-of-the-art quantum dynamics approaches in conjunction with a vibronic coupling model to simulate the excited state dynamics and the experimental TRPES of ethylene. These results show that the initial, torsional dynamics are driven by strong vibronic coupling between the ^1^*B*_1*u*_(ππ^*^) state and an optically dark ^1^*B*_3*g*_(σπ^*^) state. Significantly, this dynamical model reproduces the high-resolution VUV absorption spectrum of ethylene, a very strong indication of its validity. Based on excellent agreement between experiment and theory, we present a mechanism for the first steps in the ultrafast excited state dynamics of ethylene, with consequences for the understanding of the early time dynamics of all molecules containing carbon-carbon double bonds, namely that rapid torsional motion in unsaturated molecules is driven by strong coupling between ππ^*^ and the σπ^*^ states. Importantly, this model is consistent with previously reported trajectory-based dynamics simulations^38^ and establishes a new conceptual framework in our understanding of the photochemistry of ethylene. In addition, this model for the fundamental excited state dynamics of the carbon-carbon double bond will have broad implications for studying and controlling light-induced energy conversion, cis-trans isomerization, and photochemistry in unsaturated hydrocarbon molecules. Our results suggest that modifying the molecular geometry, for instance through chemical substitution, can influence the coupling between the relevant electronic states, and thereby affect the nonadiabatic relaxation pathways and therefore the outcome of the photochemical dynamics.

## Results

**Fig. 1:**
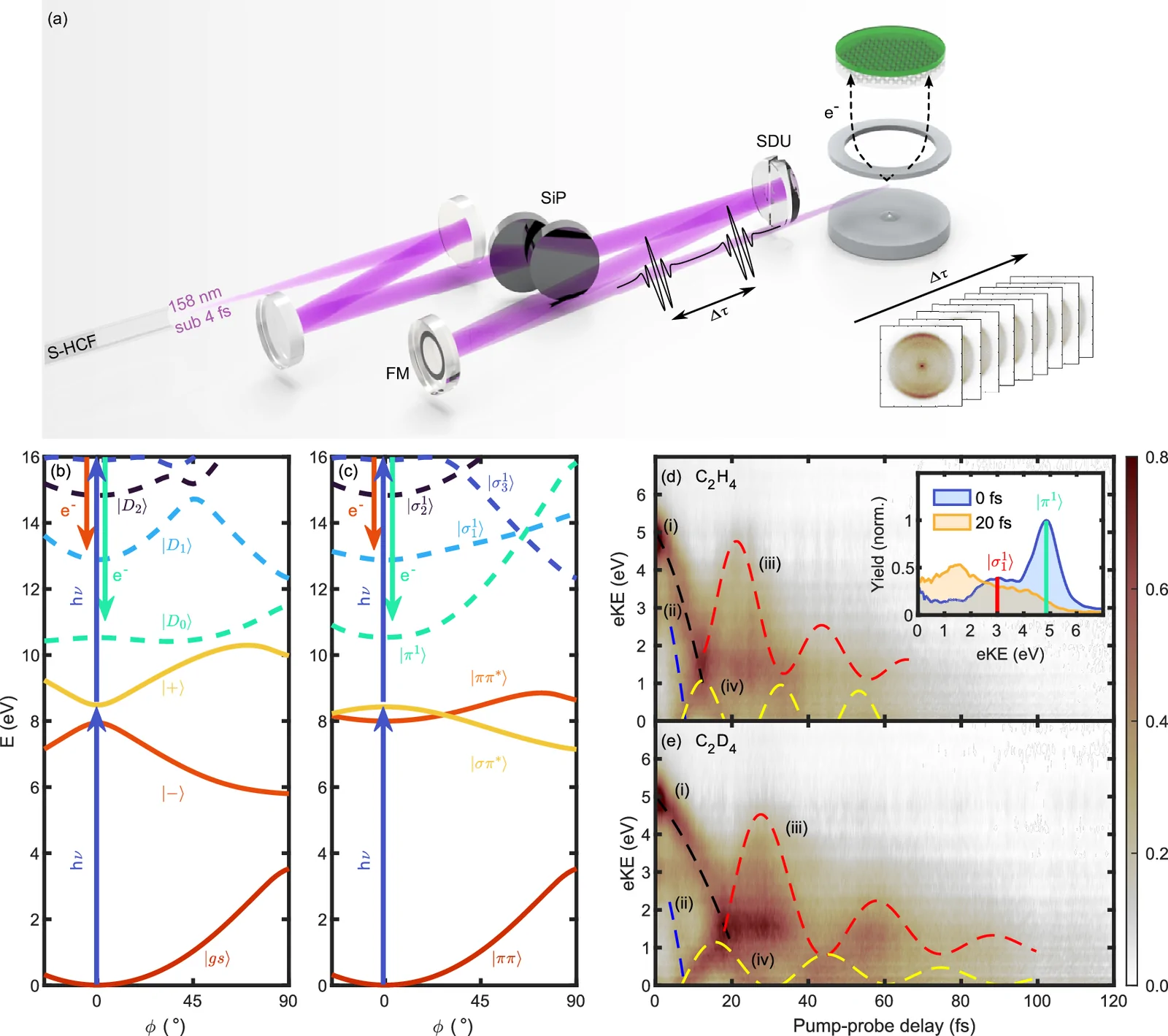
Ultrafast excited-state dynamics of ethylene probed by time-resolved photoelectron spectroscopy. **a** Layout of the experimental apparatus. The sub-4 fs VUV pulses emerging from the helium-filled capillary are first filtered by Brewster reflections from two silicon plates (SiP). Two pulse replicas are created by a split-and-delay mirror pair (SDU) and then focused into a gas jet placed inside a VMI spectrometer using a focusing mirror (FM). **b** Calculated adiabatic potential energy curves of C_2_H_4_ (solid lines) and C${}_{2}{{{{\rm{H}}}}}_{4}^{+}$ (dashed lines) along the torsional angle *ϕ*. **c** Corresponding quasi-diabatic potential energy curves obtained by a combined QD-DFT/MRCI(2) simulation (see section Computational Details). The adiabatic potential energy curves for the $\left.| -\right\rangle$ and $\left.|+\right\rangle$ states were obtained by diagonalizing the diabatic potential matrix in the $\left.| \pi {\pi }^{*}\right\rangle$ and $\left.| \sigma {\pi }^{*}\right\rangle$ state basis. In the experiment, a single VUV photon (shown in blue vertical arrow) from a first laser pulse excites the molecule to one or more intermediate electronic excited states, and a second photon from a time-delayed replica of the VUV laser pulse photoionizes the molecules leading to the emission of an electron (shown as green and red downward arrows). Time-dependent photoelectron spectra recorded in C_2_H_4_ (**d**) and C_2_D_4_ (**e**) at a pump-probe wavelength of 158 nm. The pump-probe delay-independent contributions from one-color two-photon ionization by the pump and probe pulses separately have been subtracted. The inset shows photoelectron kinetic energy spectra recorded at zero time delay (blue curve) and at a time delay of 20 fs (orange curve).

We excited ethylene with a sub-4 fs VUV pulse (see Fig. 1a and “Methods” section, along with Supplementary Information Supplementary Fig. 1a, b) at a central wavelength of 158 nm (7.85 eV), obtained via resonant dispersive wave (RDW) emission during soliton self-compression in a stretched hollow-core fiber^31,39–41^. The resulting dynamics were then probed by single photon ionization by a time-delayed sub-4 fs VUV pulse replica (see vertical blue arrows in Fig. 1b, c). At each time delay, a two-dimensional projection of the photoelectron momentum distribution resulting from (1 + 1) photoionization was recorded by a velocity map imaging (VMI) spectrometer, from which the three-dimensional photoelectron momentum distribution was reconstructed (see section Data acquisition and analysis). A typical 2D electron momentum distribution recorded at zero pump-probe delay is given as Supplementary Information Supplementary Fig. 1c, along with its corresponding angularly-integrated kinetic energy spectrum (see Supplementary Fig. 1d and inset of Fig. 1d). The spectrum recorded at zero pump-probe delay is dominated by a band centered at a photoelectron kinetic energy of 4.8 eV, attributed to single photon ionization of the optically prepared ^1^*B*_1*u*_(ππ^*^) excited state into the ground ^2^*B*_1*u*_(*π*^1^) state of the cation (green arrow in Fig. 1b), consistent with the experimentally determined vertical ionization energy of 10.95 eV^42^. A weaker photoelectron band is observed at a photoelectron kinetic energy of 3.0 eV and is assigned to single-photon ionization of the ^1^*B*_1*u*_(ππ^*^) state into the first electronically excited state of the cation, the ${}^{2}{B}_{1g}({\sigma }_{1}^{1})$ state (red arrow in Fig. 1b), with a vertical ionization energy of 12.95 eV^42^.

Time-resolved photoelectron spectra recorded in C_2_H_4_ and C_2_D_4_ are shown in Fig. 1(d) and (e). Both molecules exhibit complex behavior characterized by four main time-dependent, clearly distinguishable features, labeled (i)-(iv) in Fig. 1d, e. In feature (i) (black dashed lines in Fig. 1d, e), a very fast reduction of the photoelectron kinetic energy of the dominant contribution is observed, rapidly decreasing from 4.8 eV to around 1.5 eV within  ~ 13 fs in C_2_H_4_, and within  ~ 18 fs in C_2_D_4_. A rapid decrease in the photoelectron kinetic energy is also observed in both molecules for feature (ii) (blue dashed lines in Fig. 1d, e): from  ~ 3.0 eV at zero pump-probe delay to an energy of around  ~ 0.0 eV at a time delay of  ~7 fs. Furthermore, we observe a rapid oscillation at the highest observed photoelectron kinetic energy, feature (iii) (red dashed lines in Fig. 1d, e), with a period of 24.3 ± 0.5 fs and 30.5 ± 1.0 fs for C_2_H_4_ and C_2_D_4_, respectively, with the oscillation amplitude rapidly decaying with pump-probe time delay (see Supplementary Fig. 2 for fits). Finally, a clear delayed photoelectron band, feature (iv) (yellow dashed lines in Fig. 1d, e), appears at a pump-probe delay of  ~7 fs in both molecules and subsequently oscillates between a kinetic energy of 0.0 and 1.5 eV.

Similar behavior is observed in both C_2_H_4_ and C_2_D_4_, with a slight reduction in the initial decay rate and increases in the oscillation period for the latter. This is consistent with the expected changes of vibrational frequencies upon deuteration (see Supplementary Information and ref. ^28^). In particular, the initial decay observed in feature (i) in C_2_D_4_ is slower by a factor of close to $\sqrt{2}$ compared to C_2_H_4_, consistent with the D/H mass ratio. Feature (iii) was observed in previous experimental studies^24^, albeit with a much lower time-resolution. The photoelectron transient corresponding to feature (ii) was not previously observed. Feature (iv), which appears at low kinetic energy shortly after the zero pump-probe delay, was also not observed in ref. ^24^ due to the longer probe wavelength used in that study. We note nevertheless that when features (i) and (iv) merge at a kinetic energy of approximately 1.5 eV after a time delay of around 13 and 18 fs in C_2_H_4_ and C_2_D_4_, respectively (see Fig. 1d, e), a significant increase of the photoelectron yield is observed, which is consistent with the peak observed at around 1.4 eV by Schepp et al.^26^ in C_2_D_4_ at a time delay of around 25 fs using longer VUV laser pulse durations.

## Discussion

Previous interpretations of the early-time dynamics following ππ^*^ excitation in ethylene were formulated in terms of the topography of the corresponding adiabatic potential energy surfaces^38^. The adiabatic potential energy surface of the ^1^*B*_1*u*_(ππ^*^) state at the planar Franck-Condon point geometry has negative curvature with respect to the torsional angle (see Fig. 1b, $\left.| -\right\rangle$ state). Thus, motion along it leads to a decrease in energy. On the other hand, the ground adiabatic $\left.| {D}_{0}\right\rangle$ state of the cation increases in energy along the torsional angle. The result is that the instantaneous vertical photoionization energy increases upon torsional displacement. This is consistent with many experimental observations^24,26^, both in the present and previous works. For example, the increase in vertical ionization energy with increasing torsional angle is consistent with the oscillation of the photoelectron kinetic energy observed here. Using this interpretation, feature (iii) was previously attributed to coherent vibrational wavepacket motion in the ^1^*B*_1*u*_(ππ^*^) valence excited state along the C=C torsional mode^24^. The additional features observed here in our TRPES data, particularly feature (iv), strongly indicate that the initial excited state dynamics in ethylene cannot be fully understood solely based on the topography of the corresponding adiabatic potential energy surfaces.

Here, we introduce a minimal diabatic state representation that provides a new explanation for the origin of the initial torsional dynamics in ethylene following ππ^*^ excitation. In contrast to adiabatic states, diabatic states preserve electronic character with respect to nuclear displacements. While the definition of these states is not unique, choosing diabatic states that can be understood in terms of chemical concepts, such as simple orbital occupations, greatly aids in the interpretation of results. As detailed in the Computational Details section and the Supplementary Information, using the QD-DFT/MRCI(2) method^43–45^, quasi-diabatic potentials and couplings for the first ten electronic states were computed as a function of each vibrational coordinate, and fitted to yield a full-dimensional, ten-state model Hamiltonian (see Supplementary Fig. 5 of Supplementary Information). The potential was represented by a Taylor expansion in all coordinates except the torsional mode. As a result, the resulting model could not describe, even qualitatively, the known relaxation pathways involving coupled twist-pyramidalization and/or H-transfer processes. For this reason, we limit the analysis and discussion to the short-time dynamics that give rise to the initial torsional motion in the wave packet. As we will demonstrate later, the $\left.| \pi {\pi }^{*}\right\rangle$ state couples strongly and exclusively to the $\left.| \sigma {\pi }^{*}\right\rangle$ state by the torsional angle. The photo-physics of ethylene considered here can therefore be qualitatively described by a simple two-state representation, consisting of the strongly vibronically coupled $\left.| \pi {\pi }^{*}\right\rangle$ and $\left.| \sigma {\pi }^{*}\right\rangle$ diabatic states. The diabatic potentials for these two states are shown in Fig. 1c. At a planar geometry, *ϕ* = 0, $\left.| \pi {\pi }^{*}\right\rangle$ is the lower state, and $\left.| \sigma {\pi }^{*}\right\rangle$ the upper state, with a separation of around 0.4 eV. These two diabatic states are strongly coupled via the torsional angle, and the Hamiltonian in this two-state basis has large off-diagonal elements at twisted geometries (see Supplementary Information). Diagonalization of the corresponding 2 × 2 potential matrix gives rise to two model adiabatic potentials, denoted by *V*_−_ and *V*_+_, associated with the model adiabatic states $\left.| -\right\rangle$ and $\left.|+\right\rangle$. The model adiabatic potentials *V*_−_ and *V*_+_ are consistent with previous interpretations of the torsional dynamics in ethylene. Namely, the negative curvature of the *V*_−_ potential at the planar, *ϕ* = 0 geometry drives torsional motion. However, the interpretation in the diabatic representation is markedly different. Here, the $\left.| \pi {\pi }^{*}\right\rangle$ potential has positive curvature at the planar geometry. Instead, it is the $\left.| \sigma {\pi }^{*}\right\rangle$ state that has negative curvature. In this diabatic representation, torsional motion following ππ^*^ excitation can only occur due to strong coupling to the $\left.| \sigma {\pi }^{*}\right\rangle$ state and is driven by the rapid transfer of population to this state through vibronic coupling. As a result, a pronounced change in the electronic character of the photo-excited wavepacket, from ππ^*^ to σπ^*^, will accompany torsional motion.

To validate this interpretation and, critically, the previously overlooked strong coupling between the $\left.| \pi {\pi }^{*}\right\rangle$ and $\left.| \sigma {\pi }^{*}\right\rangle$ states, the VUV absorption spectrum of ethylene in the 6-8 eV range was first simulated using the full-dimensional, ten-state model Hamiltonian in conjunction with the multi-layer multiconfigurational time-dependent Hartree (ML-MCTDH) method^46–53^. Consistent with previous quantum dynamical studies^54^, the *a*_*u*_-symmetry torsional normal mode coordinate has been replaced with a curvilinear dihedral angle coordinate in order to better describe large amplitude twisting motion. Details of this approximation can be found in the Supplementary Information. As shown in Supplementary Fig. 8, the simulated spectrum shows excellent agreement with the high-resolution spectrum of Wu et al.^55^. Upon the removal of the coupling between the $\left.| \pi {\pi }^{*}\right\rangle$ and $\left.| \sigma {\pi }^{*}\right\rangle$ states, however, the agreement is all but lost, as shown in Supplementary Fig. 9. This confirms the strong coupling between the $\left.| \pi {\pi }^{*}\right\rangle$ and $\left.| \sigma {\pi }^{*}\right\rangle$ states which underlies it.

**Fig. 2:**
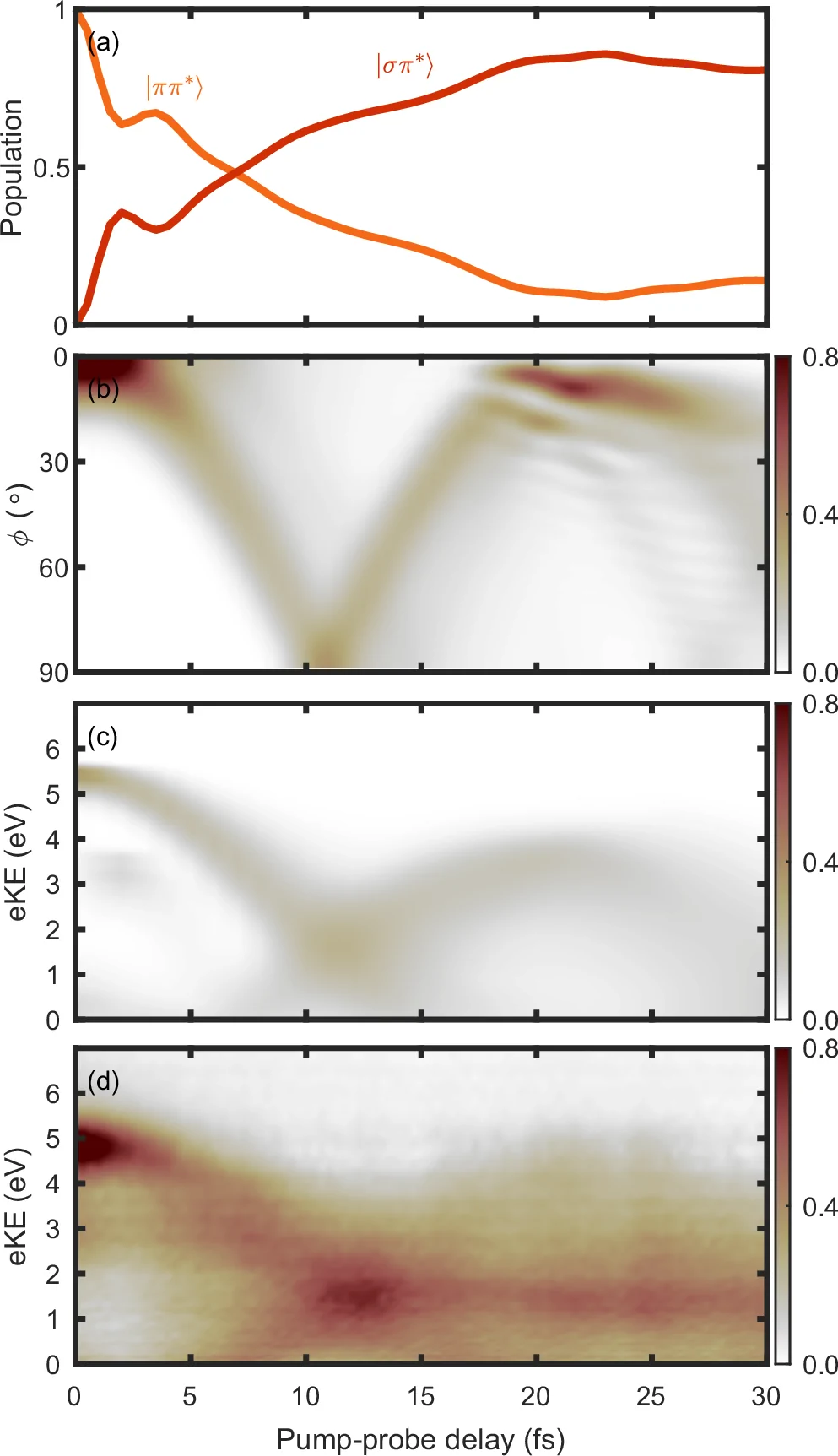
Electronic state population dynamics along with the simulated and the measured TRPES. Diabatic electronic state population dynamics resulting from MCTDH calculations using the full-dimensional ten-state model Hamiltonian in ethylene at 158 nm using vertical excitation (**a**). Zoomed-in view of the reduced density of the nuclear wavepacket *ρ*(*ϕ*, *t*) along the torsional angle *ϕ* (**b**) along with the simulated (**c**) and measured (**d**) time-dependent photoelectron spectrum at a pump-probe wavelength of 158 nm. The kinetic energy axis of the calculated TRPES was adjusted by 0.4 eV.

We now turn to the diabatic state populations computed using the full-dimensional, ten-state model Hamiltonian following vertical excitation to the $\left.| \pi {\pi }^{*}\right\rangle$ state that is populated upon excitation at 158 nm. These are shown in Fig. 2a. Importantly, we predict a rapid transfer of population to the $\left.| \sigma {\pi }^{*}\right\rangle$ state, with around 80% of the population being transferred within 25 fs. Our simulation clearly indicates that the wavepacket rapidly assumes σπ^*^ electronic character, an insight absent from all previous models. We note that no other electronic state is significantly populated within this time, strongly supporting that the early-stage dynamics in ethylene can be well described using a simplified diabatic two-state representation as discussed above. In addition, Fig. 2b shows the computed reduced nuclear density for the torsional angle, *ρ*(*ϕ*, *t*), corresponding to the probability density integrated over all degrees of freedom except *ϕ* (see Supplementary Information). The time-evolution of the torsional angle follows the population of the $\left.| \sigma {\pi }^{*}\right\rangle$ state, and further analysis shows that only the component of the wavepacket in the $\left.| \sigma {\pi }^{*}\right\rangle$ state undergoes large amplitude motion along the torsional angle (see Supplementary Fig. 7 in the Supplementary Information). Thus, it is the population of the $\left.| \sigma {\pi }^{*}\right\rangle$ state via strong coupling to the $\left.| \pi {\pi }^{*}\right\rangle$ state that gives rise to torsional motion.

We now address the question of how the change in electronic character of the wavepacket, from ππ^*^ to πσ^*^, manifests itself in the experimental TRPES. To do so, we simulated the TRPES using a perturbative model, the details of which are given in section Computational Details. The result is shown in Fig. 2c and, for comparison, the experimental result is reproduced alongside in Fig. 2d. The level of agreement is remarkable, with all major features being reproduced by our new model. In particular, the two fast-decaying photoelectron signals corresponding to features (i) and (ii) are captured, as is feature (iii), the high-energy revival appearing at later delay times. Moreover, feature (iv), the delayed onset of a low kinetic energy band at  ~ 7 fs, is also reproduced. This strongly supports our proposed model based on the strong coupling of the $\left.| \pi {\pi }^{*}\right\rangle$ and $\left.| \sigma {\pi }^{*}\right\rangle$ diabatic states.

**Fig. 3:**
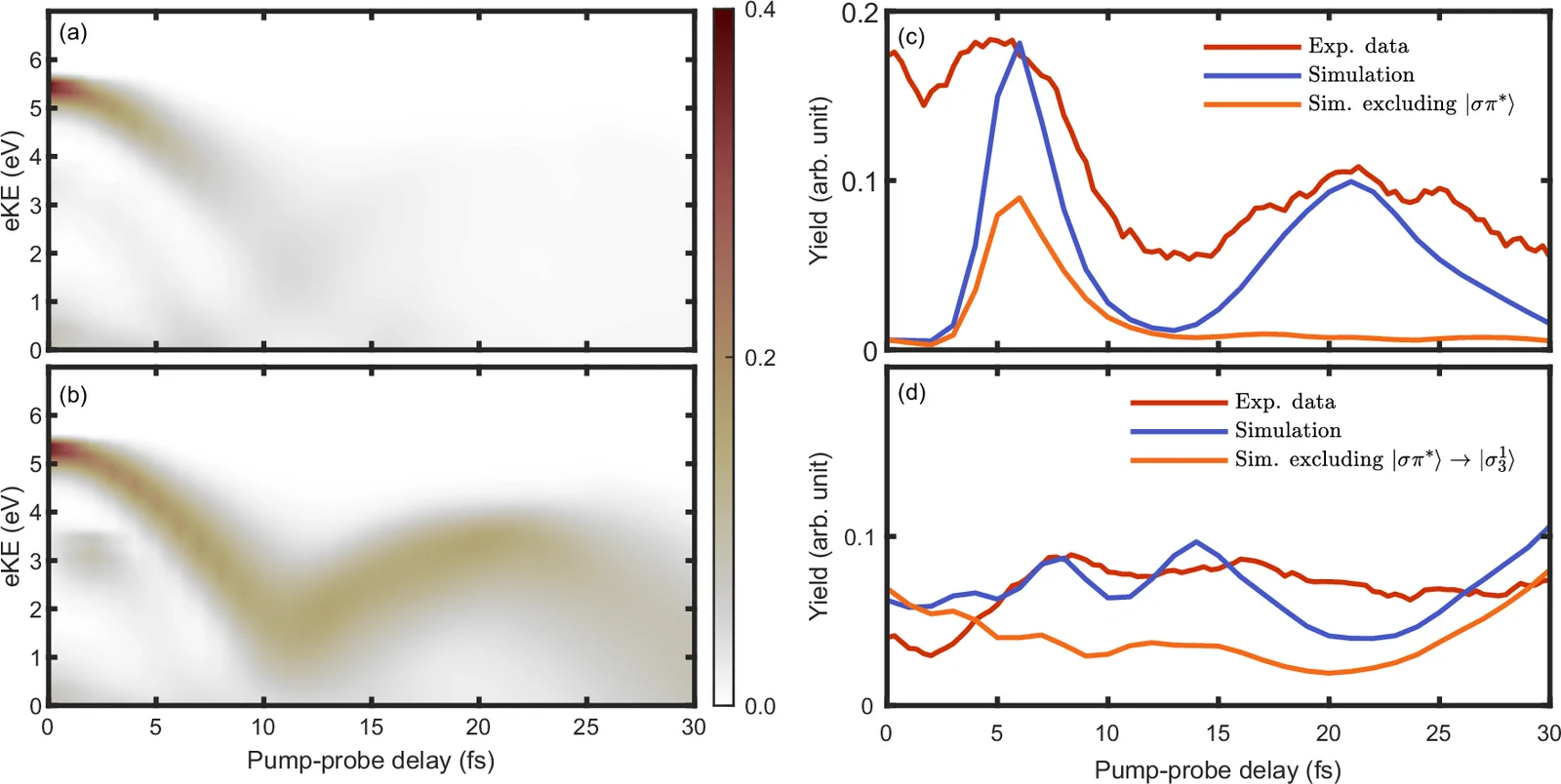
Influence of the $\left.| \sigma {\pi }^{*}\right\rangle$ state on the excited state dynamics of ethylene. Simulated TRPES excluding the contribution from the $\left.| \sigma {\pi }^{*}\right\rangle$ state (**a**). Simulated TRPES excluding the $\left.| \sigma {\pi }^{*}\right\rangle \to \left.| {\sigma }_{3}^{1}\right\rangle$ ionization channel (**b**). Comparison of experimental (red line) and simulated (blue and orange lines) time-dependent photoelectron yield integrated over the kinetic energy range 3.8–4.2 eV (**c**) and 0-0.5 eV (**d**). In (**c**), the recurrence observed at a time delay of 22 fs appears only when the $\left.| \sigma {\pi }^{*}\right\rangle$ state is included in the simulation (blue line). Similarly, in (**d**), the two peaks at  ~ 8 fs and  ~ 16 fs, corresponding to feature (iv) in the TRPES, are only present when the $\left.| \sigma {\pi }^{*}\right\rangle \to \left.| {\sigma }_{3}^{1}\right\rangle$ ionization channel is included (blue line). Simulations were performed using the full-dimensional ten-state model Hamiltonian.

This high level of agreement allows us to give a detailed interpretation of the TRPES. Firstly, features (i) and (ii), corresponding to the downward shift of the photoelectron bands centered around 4.8 and 3.0 eV within the first 10 fs, directly map onto the increase in torsional angle. The TRPES simulation shows that features (i) and (ii) correspond predominantly to the $\left.| \pi {\pi }^{*}\right\rangle \to \left.| {\pi }^{1}\right\rangle$ and $\left.| \pi {\pi }^{*}\right\rangle \to \left.| {\sigma }_{1}^{1}\right\rangle$ ionization channels, respectively. Secondly, the contribution of the $\left.| \sigma {\pi }^{*}\right\rangle$ state can be confirmed by a TRPES simulation with the $\left.| \sigma {\pi }^{*}\right\rangle$ photoionization channels artificially removed, as shown in Fig. 3a. With these ionization channels removed, the simulated and experimental TRPES no longer agree. In particular, feature (iii), corresponding to the oscillatory structure at high kinetic energies, all but vanishes. This is further shown in Fig. 3c, which displays a comparison between the simulated and measured photoelectron yield integrated over the kinetic energy interval 3.8–4.2 eV. Clearly, the oscillation of the high-energy photoelectron yield observed between 10 and 30 fs is only reproduced when the $\left.| \sigma {\pi }^{*}\right\rangle$ ionization channels are included. We therefore confidently assign this feature in the TRPES to the component of the wavepacket in the $\left.| \sigma {\pi }^{*}\right\rangle$ state.

Furthermore, feature (iv) in the TRPES, corresponding to the low kinetic energy signal appearing at a time delay of  ~ 7 fs, can also be assigned to the population of the $\left.| \sigma {\pi }^{*}\right\rangle$ state. This can be seen in Fig. 3b, which shows a TRPES simulation in which the $\left.| \sigma {\pi }^{*}\right\rangle \to \left.| {\sigma }_{3}^{1}\right\rangle$ ionization channel has been excluded, and in Fig. 3d, showing measured and simulated photoelectron yields integrated over the kinetic energy interval 0.0 to 0.5 eV. Removing this ionization channel from the TRPES simulation diminishes the photoelectron yield in this region and substantially degrades the agreement with experiment. In particular, the two maxima seen at time delays of  ~8 and  ~16 fs are only reproduced when the $\left.| \sigma {\pi }^{*}\right\rangle \to \left.| {\sigma }_{3}^{1}\right\rangle$ ionization channel is included in the simulation. Therefore, the comparison of our newly developed model with the high-time-resolution TRPES measurement clearly confirms the change in electronic character along the torsional coordinate, an aspect that, to the best of our knowledge, has not been discussed and was overlooked in earlier studies.

We have developed an important model that sheds new light on the early-time dynamics of ethylene following excitation to the $\left.| \pi {\pi }^{*}\right\rangle$ state, by combining state-of-the-art TRPES experiments performed with sub-4 fs VUV pulses and ab initio quantum dynamics simulations. Importantly, our study demonstrates that the $\left.| \sigma {\pi }^{*}\right\rangle$ state plays a critical role in the initial excited state dynamics of the carbon-carbon double bond. In particular, we show that torsional motion is driven by the coupling between the $\left.| \pi {\pi }^{*}\right\rangle$ and the $\left.| \sigma {\pi }^{*}\right\rangle$ states and that the photoexcited wavepacket undergoes a change from ππ^*^ to σπ^*^ electronic character within less than 10 fs. The coupling of the $\left.| \pi {\pi }^{*}\right\rangle$ and $\left.| \sigma {\pi }^{*}\right\rangle$ states represent a general but, until now, unappreciated phenomenon, with important implications for understanding ultrafast isomerization dynamics in molecules, particularly those involving C=C double bonds. We expect that a deeper understanding of the coupled electronic-nuclear dynamics occurring at the C=C bond in polyenes could enable improved control over the underlying processes responsible for ultrafast isomerization dynamics in this class of molecules. Finally, our work highlights the importance of performing experiments at high temporal resolution in combination with advanced quantum dynamics simulations of the experimental observables using the QD-DFT/MRCI(2) coupled with the multi-layer multiconfigurational time-dependent Hartree (ML-MCTDH) approach: it permits fully resolving the ultrafast electronic-vibrational dynamics in excited molecules, where complex non-adiabatic coupling typically occurs between multiple electronic states along multiple reaction coordinates.

## Methods

### Experiment

The experiment was conducted using a commercial Ti:Sa laser amplifier that provided 2 mJ, 37 fs pulses at a central wavelength of 800 nm and a repetition rate of 1 kHz. The pulses were first spectrally broadened inside a 3-meter-long stretched hollow-core fiber (∅=450 μm) filled with 120 mbar of argon in a pressure gradient configuration, thus generating 10 fs near-infrared laser pulses. These pulses were then directed into a second, 1.5 m long, stretched hollow-core fiber (∅=250 μm) filled with approximately 1.2 bar of helium, where tunable VUV pulses were generated via resonant dispersive wave emission^31,39^. Fine-tuning of the central wavelength of the VUV pulse at the output of the second fiber was achieved by adjusting the pressure and input energy of the NIR laser beam. After passing through the second hollow-core fiber, the broadband NIR laser pulse was filtered out from the VUV pulse using two Brewster-angled silicon plates at an angle of incidence of 75^∘^. A maximum VUV pulse energy of 1.5 μJ was measured after the Brewster angle silicon plates. The VUV pulse was then split into two using a custom-built split-and-delay mirror unit, composed of two D-shaped aluminum-enhanced mirrors. One of the D-shaped mirrors was mounted on a piezo-motorized delay stage to precisely adjust the delay between the two pulses. The two pulses were propagated through a few mbar He atmosphere and focused into a continuous jet of C_2_H_4_ or C_2_D_4_ molecules at the center of a VMI spectrometer. Prior to entering the VMI chamber, the VUV beams passed through three apertures, which were used for differential pumping and to remove most scattered VUV photons. The molecular beam was produced by expanding less than 100 mbar of ethylene through a 100 μm diameter nozzle connected to the repeller plate of the VMI spectrometer. The resulting photoelectrons were accelerated toward a dual micro-channel plates/phosphor screen assembly, and the phosphorescence was recorded using a CCD camera. Before conducting any experiments, the spectrum of the VUV pulse was recorded using a commercial VUV spectrometer (McPherson 234/302 spectrograph equipped with 1200 lines/mm Al+MgF_2_-coated flat-field grating and a CCD array (Andor model DU920N)) (see Supplementary Fig. 1a). The pulse duration was characterized by recording the ionization yield resulting from two-photon ionization of krypton as a function of the delay between the two VUV pulses. A typical autocorrelation measurement recorded at a pump wavelength of 158 nm is shown in the Supplementary Fig. 1b of the Supplementary Information. The autocorrelation recorded in krypton was well-reproduced by a convolution of two Gaussian pulses with a FWHM of 3.7 ± 0.3 fs.

### Data acquisition and analysis

Photoelectron momentum distributions were recorded in steps of 0.33 fs in a delay range between –200 fs and 200 fs for C_2_D_4_ and –133 fs and 133 fs for C_2_H_4_. Each momentum distribution was integrated over 1000 laser shots. A total of four temporal scans were recorded and then averaged for all measurements. To recover the 3D momentum distribution from the 2D momentum distribution measured with the VMI spectrometer, an Abel inversion based on Legendre decomposition was applied^56^. Exploiting the cylindrical symmetry of the velocity-map imaging (VMI) images, each image was symmetrized by averaging its four quadrants before inversion. This procedure improved the signal-to-noise ratio while preserving the underlying photoelectron distribution. All subsequent analyses were performed using the symmetrized images. In the Legendre decomposition, the signal was expressed as:

1 $$S({E}_{k},\theta,t)=\sigma ({E}_{k},t)\left[1+{\beta }_{2}\left.({E}_{k},\theta,t)\right){{{{\rm{P}}}}}_{2}(\cos \theta )+{\beta }_{4}({E}_{k},\theta,t){{{{\rm{P}}}}}_{4}(\cos \theta )\right],$$

with *σ*(*E*_*k*_, *t*), the angularly-integrated ionization cross-section, *β*_*n*_, *n* = 2 and 4, the well-known angular asymmetry parameters characterizing the photoelectron angular distribution (PAD) for a two-photon ionization process, and ${{{{\rm{P}}}}}_{n}(\cos \theta )$, the Legendre polynomials of order n.

We note that because the experiment employed two pulse replicas, the measured TRPES was symmetric with respect to zero time delay, such that the signals recorded at positive and negative delays were identical within the experimental uncertainty. To further improve the signal-to-noise ratio, the data acquired at positive and negative time delays were therefore averaged.

### Computational details

The TRPES for the 158 nm pump-probe wavelength was simulated using wavepacket propagations performed using the ML-MCTDH method as implemented in the Heidelberg MCTDH package^57^. The Hamiltonian was represented in a diabatic state basis consisting of the first ten neutral and four cation states, and all twelve nuclear degrees of freedom were included. The nuclear coordinates used correspond to the torsional angle *ϕ* and the eleven non-*a*_*u*_ normal modes. The diabatic potential matrix was represented using a truncated fourth-order Taylor expansion in terms of the non-*a*_*u*_ normal modes, with only the one-mode terms retained, corresponding to a vibronic coupling Hamiltonian approximation^58,59^. The on-diagonal diabatic potential matrix elements along the torsional angle were expanded in terms of basis functions ${\sin }^{2}(n\phi )$, *n* = 1, …, 9, and the off-diagonal elements in terms of the basis functions $\sin ([2n-1]\phi )$, *n* = 1, …, 5, which obey both the correct point group symmetries and periodic boundary conditions for this degree of freedom. The expansion coefficients for the neutral state block of the diabatic potential matrix were determined by least squares fitting to quasi-diabatic potentials computed at the QD-DFT/MRCI(2)/aug-cc-pVTZ level of theory using the QE8 Hamiltonian^60^. For the cation state block, the expansion coefficients for the normal mode coordinates were taken from the previously reported quadratic vibronic coupling Hamiltonian of Hazra and Nooijen^61^, while the expansion coefficients for the torsional angle part of the potential were fitted to the results of QD-DFT/MRCI(2)/aug-cc-pVTZ calculations. All QD-DFT/MRCI(2) calculations were performed using the GRaCI software package^62^. The TRPES was simulated using the perturbative procedure developed by Richings and Worth^63^, in which the signal at a pump-probe delay *Δt* is approximated by the vertical projection of the pumped wavepacket propagated to time *t* = *Δt* onto the cation manifold, followed by calculation of the Fourier transform of the autocorrelation function of the subsequently propagated wavepacket. The full details of these calculations are given in the Supplementary Information.

## Supplementary information

Supplementary Information

Transparent Peer Review file

## Data Availability

The data underlying the figures are available in the Figshare repository: 10.6084/m9.figshare.32880398.

## Code availability

All QD-DFT/MRCI(2) calculations were performed using the GRaCI software package available at: https://github.com/schuurman-group/graci.git. The analysis code used to generate the data presented in this study is available from the corresponding authors upon request.
