# Supplementary material for: Few-femtosecond photoelectron spectroscopy reveals fundamental photodynamics of the carbon-carbon double bond in ethylene
Source: Nat Commun. 2026 Jul 23;17:8991. doi: 10.1038/s41467-026-75782-3 (PMC13507108; doi:10.1038/s41467-026-75782-3)
Supplement: Supplementary file 1 [file 41467_2026_75782_MOESM1_ESM.pdf]

Few-Femtosecond Photoelectron Spectroscopy Reveals Fundamental Photodynamics of the Carbon–Carbon Double Bond in Ethylene: Supplementary Information

Arnab Sen[1†], Simon P. Neville[2†], Martin Kretschmar[1†], Martha Yaghoubi Jouybari[3†], Rostyslav Danylo[1], José R. C. Andrade[1], Marc J. J. Vrakking[1], Albert Stolow[2,3,4,5*], Tamas Nagy[1], Michael S. Schuurman[2,3*], Arnaud Rouzée[1*]

[1]Max-Born-Institut, Max Born Strasse 2A, Berlin, 12489, Germany.
[2]National Research Council Canada, 100 Sussex Drive, Ottawa, K1A 0R6, Ontario, Canada.
[3]Department of Chemistry and Biomolecular Sciences, University of Ottawa, 10 Marie Curie, Ottawa, K1N 6N5, Ontario, Canada.
[4]Department of Physics, University of Ottawa, 150 Louis Pasteur, Ottawa, K1N 6N5, Ontario, Canada.
[5]NRC-uOttawa Joint Centre for Extreme Photonics, University of Ottawa, 25 Templeton St., Ottawa, K1N 6N5, Ontario, Canada.

*Corresponding author(s). E-mail(s): astolow@uottawa.ca; michael.schuurman@uottawa.ca; rouzee@mbi-berlin.de;
†These authors contributed equally to this work.

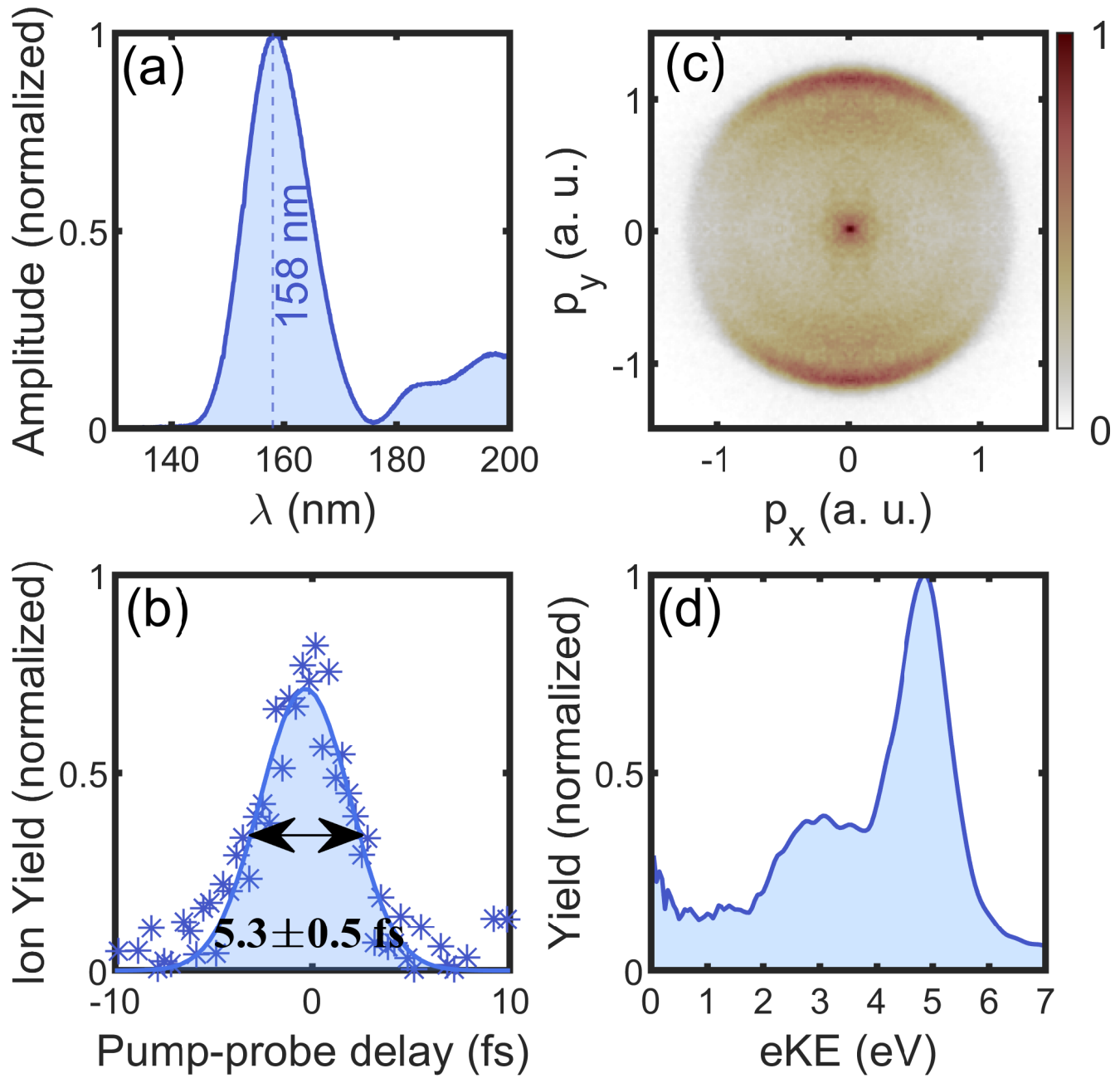

Suppl. Fig. 1: (a) Measured VUV spectrum at a central wavelength of 158 nm. (b) Autocorrelation signal recorded in krypton. (c) 2D photoelectron momentum distribution recorded in C_2H_4 at time overlap of the two 158 nm laser pulses. Delay-independent contributions from one-color two-photon ionization by the individual pump and probe pulses have been subtracted. (d) Corresponding photoelectron kinetic energy spectrum measured at time overlap.

Supplementary Note 1: Experimental Details

The spectrum of the VUV pulses generated through resonant dispersive wave emission in a stretched hollow-core fiber was characterized using a commercial VUV spectrometer. The recorded spectrum, centered at 158 nm, is shown in Suppl. Fig. 1(a). The pulse duration of the VUV pulse was further determined by monitoring the two-photon photoionization yield of krypton as a function of the delay between the two VUV pulse replica [1]. The measured autocorrelation trace obtained in krypton is presented in Suppl. Fig. 1(b). Based on this autocorrelation, we estimated a pulse duration of 3.7±0.3 fs.

The 2D photoelectron momentum distribution of C_2H_4, recorded at the temporal overlap of the two 158 nm laser pulses, is shown in Suppl. Fig. 1(c), together with its corresponding angularly-integrated photoelectron spectrum (see Suppl. Fig. 1(d)).

Supplementary Note 2: Quantitative analysis of TRPES

A quantitative analysis of the TRPES at each kinetic energy using fits based on exponential decay functions combined with periodic components, as performed in [2, 3], is challenging due to the significant variation in the photoelectron kinetic energy spectrum as a function of time delay observed experimentally. To address this, we analyzed the time evolution of the photoelectron yield within two specific kinetic energy (KE) ranges, rather than attempting a point-by-point fit across the entire spectrum. At a probe wavelength of 158 nm, the selected KE ranges were: (1) 0.0–1.0 eV, representing the low-energy region, and (2) 2.5–6.0 eV, representing the high-energy region. The time-dependent photoelectron yields within these energy intervals were then fitted using a combination of exponential rise/decay functions and periodic modulations using the following expressions:

$$S_1(t) = A_1 \times \exp\left(\frac{-t}{\tau_1}\right) + A_2 \times \cos\left[\frac{2\pi t}{T} + \phi_1\right] \times \exp\left(\frac{-t}{\tau_2}\right), \qquad \text{(Suppl. Eq. 1)}$$

$$S_2(t) = A_1 \times \left(\exp\left(\frac{-t}{\tau_1}\right) - \exp\left(\frac{-t}{\tau_2}\right)\right) + A_2 \times \cos\left[\frac{2\pi t}{T} + \phi_1\right] \times \exp\left(\frac{-t}{\tau_3}\right). \qquad \text{(Suppl. Eq. 2)}$$

In equation Suppl. Eq. 1, the first term represents an exponential decay characterized by amplitude A_1 and decay time τ_1. The second term describes a damped

oscillation, with amplitude A_2, oscillation period T, phase ϕ_1, and damping time τ_2. In equation Suppl. Eq. 2, the first term describes a bi-exponential transient that starts from zero at $t = 0$, rises initially with the characteristic time τ_2, and then decays with time constant τ_1. The amplitude A_1 scales the overall magnitude of this contribution. This bi-exponential function is used to account for a possible additional ionization channel that will be delayed with respect to time overlap. The second term is used to account for the oscillations observed in the low-energy photoelectron yield, characterized by an amplitude A_2, an oscillation period T, a phase ϕ_1, and a damping time τ_3.

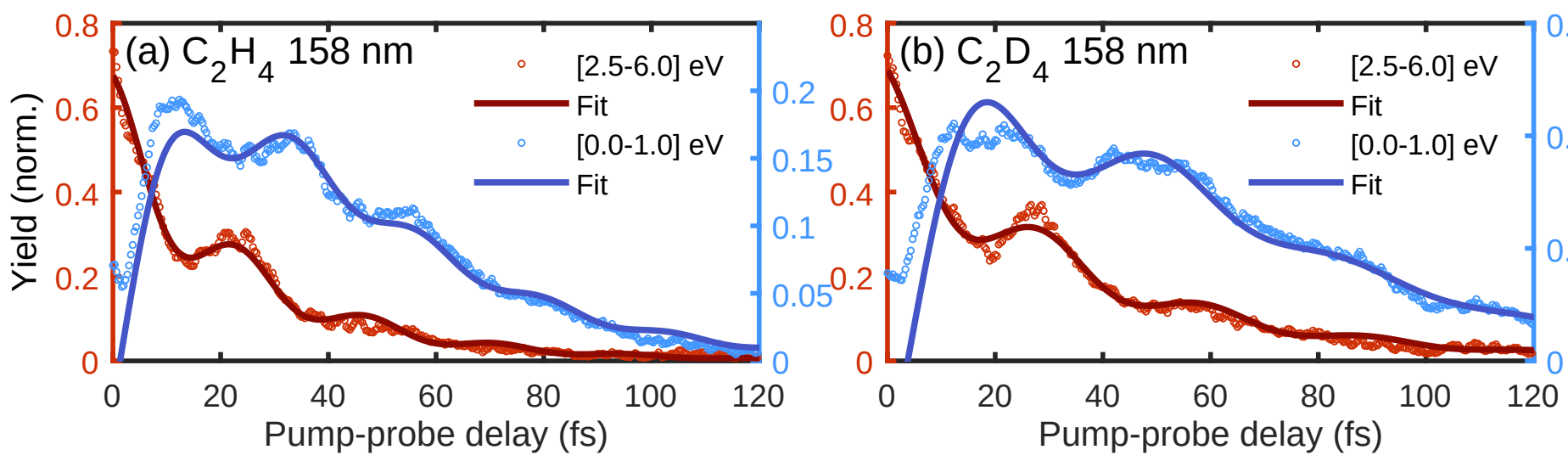

Suppl. Fig. 2: Time-dependent integrated photoelectron yields in the kinetic energy ranges given by [0.0-1.0] eV (blue circles) and [2.5-6.0] eV (red circles) for C_2H_4 (a) and C_2D_4 (b).

Suppl. Fig. 3(a)–(b) shows the time-dependent integrated photoelectron yields for C_2H_4 and C_2D_4 at 158 nm probe wavelength, within the kinetic energy ranges described above, along with the corresponding fit results. Our simple model allows us to reproduce the observed dynamics very well in the two aforementioned kinetic energy ranges. The extracted fitting parameters are provided in Suppl. Table 1 and Suppl. Table 2. In ethylene (C_2H_4), the photoelectron yield in the high kinetic energy region exhibits a rapid decay, characterized by a time constant of 25.9±0.5 fs. For its isotopologue, C_2D_4, the decay is slightly slower, with a time constant of 37.4±1.0 fs. These values closely match previous results obtained at 160 nm [4, 5], which reported excited-state lifetimes of 25±3 fs for C_2H_4 and 38±4 fs for C_2D_4. The decay constant

retrieved for C_2H_4 is also consistent with the single-exponential decay time of 32±2 fs measured in [2] and 21±4 fs measured in previous time-resolved ion mass spectroscopy experiments [6, 7].

Pronounced oscillations in the photoelectron yield are observed superimposed on the fast decay, with a beating frequency of 1240 cm^{-1} (corresponding to a period T_1=24.3±0.5 fs) at 158 nm in C_2H_4. In C_2D_4, similar oscillatory components are detected at 1093 cm^{-1} (T=30.5±1.0 fs). These oscillations are in close agreement with earlier studies [6] and were attributed to nuclear wavepacket dynamics on the adiabatic electronic excited state associated to the $^1B_{1u}(\pi\pi^*)$ state. Specifically, they are generally associated with the torsional mode, the C=C stretching vibration, and the symmetric scissoring motion, which are the primary vibrational modes activated upon excitation to the $^1B_{1u}(\pi\pi^*)$ excited state [8].

Interestingly, the effect of deuteration on the normal modes initially populated upon excitation was investigated in [5]. The expected influence of deuteration on the vibrational dynamics can be estimated from the ratio of reduced masses, which affects the vibrational frequency as:

$$\frac{\omega_D}{\omega_H} = \sqrt{\frac{\mu(m_{\mathrm{H}}, m_{\mathrm{C}})}{\mu(m_{\mathrm{D}}, m_{\mathrm{C}})}} \qquad \text{(Suppl. Eq. 3)}$$

This leads to approximate ratios of 1.41, 1.38, and 1.30 for the torsional, pyramidalization, and symmetric stretch modes, respectively. In our measurements, the ratio of the vibrational period observed in ethylene and its deuterated isotopologue is close to $\sqrt{2}$, and is therefore in agreement with the expected change of the vibrational frequency upon deuteration for the torsional mode.

In the low KE range, the photoelectron yield exhibits a rapid rise before decaying slowly with a time constant of 23±2 fs in ethylene. Pronounced oscillations in the photoelectron yield are also observed, exhibiting frequencies very close to those detected

in the higher kinetic energy range (see Suppl. Table 1 and Suppl. Table 2). Notably, these oscillations are out of phase.

**Parameters**	**λ: 158 nm**	
	C_2H_4	C_2D_4
A_1	0.54	0.56
τ_1 (fs)	25.9±0.5	37.4±1.0
A_2	0.13	0.13
T (fs)	24.3±0.5	30.5±1.0
ϕ (rad)	-0.15	0.08
τ_2 (fs)	25.0±2.5	31.8±3.0

Suppl. Table 1: Fitted parameters obtained from a least-squares fit of the photoelectron yield integrated over the [2.5-6.0] eV kinetic energy range using Suppl. Eq. 1.

**Parameters**	**λ: 158 nm**	
	C_2H_4	C_2D_4
A_1	4.6	2
τ_1 (fs)	23±2	33.5±1.0
τ_2 (fs)	20.8±1.0	25.6±2.0
A_2	0.032	0.09
T (fs)	23.4±1.0	35.3±2.0
ϕ (rad)	-2.8	-3.08
τ_3 (fs)	37±5	30±2

Suppl. Table 2: Fitted parameters obtained from a least-squares fit of the photoelectron yield integrated over the [0.0-1.0] eV kinetic energy range using Suppl. Eq. 2.

Supplementary Note 3: Analysis of photoelectron angular distributions (PADs)

We have analyzed the photoelectron angular distributions (PADs) extracted from the VMI measurements and evaluated the corresponding anisotropy parameters, β_2 and β_4, which characterize the angular distribution of photoelectrons generated in the two-photon ionization process. Small variations in these parameters are indeed observed (see Suppl. Fig. 3(a) and (b) below). Nevertheless, the observed differences remain relatively subtle and do not allow for an unambiguous assignment of the photoelectron signal to either the $\pi\pi^*$ or $\sigma\pi^*$ state.

This behavior can be rationalized using symmetry considerations. For ethylene near the Franck–Condon geometry, where the molecular symmetry is described by D_{2h}, the symmetry-allowed ionization transition must satisfy

$$\Gamma^{\text{excited}} \otimes \Gamma^{\mu} \otimes \Gamma^{\text{cation}} \otimes \Gamma^{\text{free electron}} = A_{1g}, \qquad \text{(Suppl. Eq. 4)}$$

where Γ^{excited}, Γ^{μ}, Γ^{cation}, and $\Gamma^{\text{free electron}}$ denote the irreducible representations of the excited electronic state, dipole operator, ionic state, and outgoing photoelectron continuum, respectively.

The $\pi\pi^*$ state of ethylene possesses B_{2u} symmetry at the Franck–Condon geometry (see section Supplementary Note 6: Analysis of the ground state VUV absorption spectrum for the assigned symmetries of the neutral and ionic states at the Franck–Condon point), whereas the $\sigma\pi^*$ state has B_{2g} symmetry. At the Franck-Condon point, the photoelectron partial wave decomposition for the $|\pi\pi^*\rangle \to |\pi^1\rangle$ ionization channel is expected to contain p and f angular momentum whereas for $|\sigma\pi^*\rangle \to |\pi^1\rangle$ ionization channel, it is expected to contain s and d partial wave. This is in agreement with our experimental observation. At the zero delay, the photoelectron associated with the $|\pi\pi^*\rangle \to |\pi^1\rangle$ ionization channel peaks along the laser polarization

(β_2 ~1.4) as expected for a $p-$wave like electronic wavefunction. At a later time, around ~22 fs, the photoelectron signal observed at ~4.3 eV is assigned to the $|\sigma\pi^*\rangle \rightarrow |\pi^1\rangle$ ionization channel following the return of the vibronic wavepacket to the Franck-Condon region. In this case, the measured anisotropy parameter decreases to $\beta_2 \sim 0.8$, substantially smaller than the value observed at zero delay, where ionization from the $|\pi\pi^*\rangle$ state dominates. This reduction is likewise consistent with the expected s- and d-wave character of the $|\sigma\pi^*\rangle \rightarrow |\pi^1\rangle$ ionization channel.

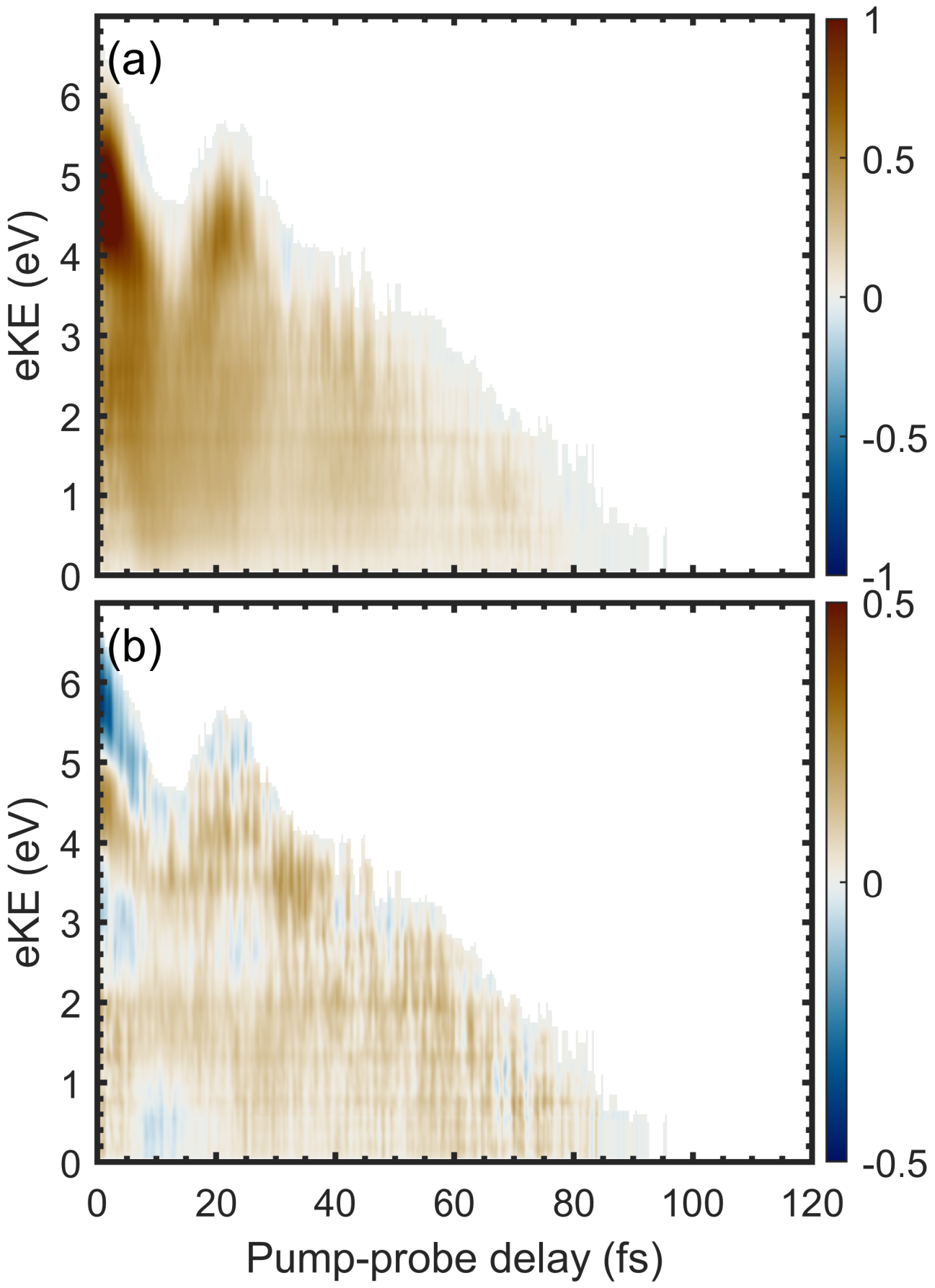

Suppl. Fig. 3: Time- and energy-resolved photoelectron angular anisotropy parameters of C_2H_4: (a) $\beta_2(E_k, t)$ and (b) $\beta_4(E_k, t)$.

However, following excitation to the $\pi\pi^*$ state, ethylene undergoes substantial torsional motion, which drives the molecule away from the equilibrium D_{2h} geometry. As the torsional angle approaches 90°, the molecular symmetry evolves toward D_{2d} through intermediate D_2 geometries, reducing the applicability of strict D_{2h} selection rules. In D_2 symmetry, due to the loss of mirror-plane symmetry and inversion symmetry, both the $\pi\pi^*$ and $\sigma\pi^*$ states correlate to the same B_2 irreducible representation and therefore become symmetry-indistinguishable. Consequently, outside of the equilibrium geometry, it is no longer possible to distinguish whether the ionization is taking place from the $\pi\pi^*$ or $\sigma\pi^*$ state solely based on their PADs. In conclusion, an unambiguous assignment of the photoelectron signal to either electronic state using PADs alone is not straightforward.

Supplementary Note 4: Quantum dynamics simulations

a. Model Hamiltonian

The field-free Hamiltonian $\hat{H}$ was represented in the basis $\{|i^{(n)}\rangle\} \cup \{|i^{(c)}\rangle\}$ of neutral and cation diabatic electronic states $|i^{(n)}\rangle$ and $|i^{(c)}\rangle$, respectively. As the two sets of states are not coupled by the field-free Hamiltonian, its representation in this basis takes the following block diagonal form:

$$\boldsymbol{H} = \boldsymbol{H}^{(n)} \oplus \boldsymbol{H}^{(c)}, \quad \text{(Suppl. Eq. 5)}$$

$$H_{ij}^{(n)} = \langle i^{(n)}|\hat{H}|j^{(n)}\rangle, \quad \text{(Suppl. Eq. 6)}$$

$$H_{ij}^{(c)} = \langle i^{(c)}|\hat{H}|j^{(c)}\rangle. \quad \text{(Suppl. Eq. 7)}$$

The Hamiltonian matrix $\boldsymbol{H}$ is nuclear-coordinate dependent owing to the parametric dependence of the electronic states on the nuclear coordinates. Thus, to proceed, our choice of nuclear coordinates must be specified. As we are interested in the short-time dynamics following photo-excitation, a natural choice would usually be the dimensionless mass- and frequency-scaled ground state normal modes Q_α. This is an excellent and convenient choice when only small-amplitude nuclear motion takes place. However, even on the timescale of a couple of tens of fs, there exists a large torsional motion in ethylene, particularly following excitation to the $|\pi\pi^*\rangle$ state, and to describe this using of rectilinear normal mode coordinates is highly complicated. We thus choose to make the following approximation. The single a_u out-of-plane CH_2 twisting normal mode is replaced with the curvilinear torsional mode ϕ, defined as the angle between the two vectors connecting the hydrogen atoms within each CH_2 group. As shall be detailed, this necessitates the introduction of some approximations into the construction of the nuclear kinetic energy operator. However, the ease with which the large-amplitude torsional motion following excitation to the $\pi\pi^*$ state can be described makes this coordinate substitution highly desirable. We note that such a coordinate set has been used before by Cattarius *et al.* in their study of the spectroscopy and dynamics of the butatriene cation [9].

To proceed, we partition the blocks $\boldsymbol{H}^{(b)},\ b = n, c$ into nuclear kinetic energy and potential energy contributions,

$$\boldsymbol{H}^{(b)}(\bar{\boldsymbol{Q}}, \phi) = \boldsymbol{T}^{(b)}(\bar{\boldsymbol{Q}}, \phi) + \boldsymbol{W}^{(b)}(\bar{\boldsymbol{Q}}, \phi), \qquad b = n, c, \qquad \text{(Suppl. Eq. 8)}$$

where $\bar{\boldsymbol{Q}}$ denotes the vector of non-a_u normal modes.

The nuclear kinetic energy contribution reads as follows:

$$\boldsymbol{T}^{(b)}(\bar{\boldsymbol{Q}}, \phi) = \left[-\frac{1}{2} \sum_{\alpha} \omega_\alpha \frac{\partial^2}{\partial \bar{Q}_\alpha^2} - \frac{1}{m_H} \nu(\bar{\boldsymbol{Q}}) \frac{\partial^2}{\partial \phi^2} \right] \mathbf{1}, \qquad \text{(Suppl. Eq. 9)}$$

where ω_α is the frequency corresponding to the non-a_u normal mode $\bar{Q}_\alpha$, m_H is the mass of the hydrogen atom, and $\nu(\bar{\boldsymbol{Q}})$ is a function of the non-a_u normal modes that derives from the corresponding G-matrix element for the torsional angle ϕ (see Reference 10 for details). This term is not in the sum-of-products form required for use in MCTDH calculations. To proceed, we approximate the torsional angle G-matrix element by its Franck-Condon point value, leading to the following approximate, sum-of-products-form nuclear kinetic energy operator:

$$\boldsymbol{T}^{(b)}(\bar{\boldsymbol{Q}},\phi) \approx \left[-\frac{1}{2}\sum_{\alpha} \omega_\alpha \frac{\partial^2}{\partial \bar{Q}_\alpha^2} - \frac{1}{6.07 m_H}\frac{\partial^2}{\partial \phi^2} \right] \mathbf{1}, \qquad \text{(Suppl. Eq. 10)}$$

where atomic units are assumed.

The potential contributions, $\boldsymbol{W}^{(b)}(\bar{\boldsymbol{Q}},\phi)$, are approximated in the spirit of the vibronic coupling Hamiltonian approach of Köppel, Domcke, and Cederbaum[11, 12] by low-order expansions of the diabatic potential matrix elements about the Franck-Condon point [13]. For the non-a_u normal modes $\bar{Q}_\alpha$, a 4th-order Taylor expansion was used, corresponding exactly to a vibronic coupling Hamiltonian within this subspace. For the torsional angle ϕ, an expansion that obeys the boundary conditions $\boldsymbol{W}^{(b)}(\phi = -\pi) = \boldsymbol{W}^{(b)}(\phi = \pi)$ must be used. Additionally, due to the point group symmetry of the problem, the off-diagonal potential matrix elements that are non-zero by symmetry, i.e., those between the $\pi 3s/\sigma 3s$ and the $\pi\pi^*/\sigma\pi^*$ states, have odd symmetry with respect to ϕ, whilst all on-diagonal potential matrix elements have even symmetry with respect to ϕ. To satisfy these requirements, the on-diagonal potential matrix elements were expanded in terms of the basis functions $\sin^2(n\phi)$, $n = 1, \ldots, 9$, and the off-diagonal elements in terms of the basis functions $\sin([2n-1]\phi)$, $n = 1, \ldots, 5$. Thus, our model diabatic potential matrices read as follows:

$$W_{ii}^{(b)}(\bar{\boldsymbol{Q}},\phi) \approx E_i + \sum_{n=1}^{4}\sum_{\alpha} \tau_{\alpha n}^{(i,i)} \bar{Q}_\alpha^n + \sum_{n=1}^{9} a_n^{(i)} \sin^2(n\phi), \qquad \text{(Suppl. Eq. 11)}$$

$$W_{ij}^{(b)}(\bar{\boldsymbol{Q}}, \phi) \approx \sum_{n=1}^{4}\sum_{\alpha} \tau_{\alpha n}^{(i,j)} \bar{Q}_{\alpha}^{n} + \sum_{n=1}^{5} b_n^{(i,j)} \sin([2n-1]\phi), \qquad i \neq j, \qquad \text{(Suppl. Eq. 12)}$$

where, E_i denotes, depending on the block b, the vertical excitation or ionization energy of the electronic state $|i^{(b)}\rangle$ at the Franck-Condon point.

b. Simulation of the ground state VUV absorption spectrum

The ground state VUV absorption spectrum, $I(E)$, was computed via the Fourier transforms of wavepacket autocorrelation functions for initial states corresponding to vertical excitation to each of the optically bright valence electronic states:

$$\begin{aligned} I(E) &\sim \sum_{i^{(n)}} \int_{-\infty}^{\infty} a_{i^{(n)}}(t) \exp(iEt) dt \\ &= 2Re \sum_{i^{(n)}} \int_{0}^{\infty} a_{i^{(n)}}(t) \exp(iEt) dt, \end{aligned} \qquad \text{(Suppl. Eq. 13)}$$

where the sum is restricted to the bright electronic states. Here, $a_{i^{(n)}}(t)$ is the wavepacket autocorrelation function computed following vertical excitation of the ground vibronic state $|\Phi_0\rangle$ to the electronic state $|i^{(n)}\rangle$:

$$a_{i^{(n)}}(t) = \langle \Phi_0 | \hat{\epsilon}_{i^{(n)}} \exp(iEt) \hat{\epsilon}_{i^{(n)}} | \Phi_0 \rangle, \qquad \text{(Suppl. Eq. 14)}$$

$$\hat{\epsilon}_{i^{(n)}} = \left\{ |i^{(n)}\rangle\langle 0^{(n)}| + h.c. \right\}. \qquad \text{(Suppl. Eq. 15)}$$

In practice, a finite wavepacket propagation time T is used. To minimize the numerical artifacts that result from this, the integrand in Equation Suppl. Eq. 18 was multiplied by the following window function:

$$G(t) = \cos^2\left(\frac{\pi t}{2T}\right) \Theta(t - T). \qquad \text{(Suppl. Eq. 16)}$$

In order to account for the phenomenological broadening present in the experimental spectrum, the integrand was further multiplied by the damping function

$$F(t) = \exp\left(-\frac{t}{\tau}\right), \qquad \text{(Suppl. Eq. 17)}$$

where the value of the damping time τ controls the phenomenological broadening. Specifically, the effect of multiplication of the autocorrelation function by $F(t)$ is to convolute the spectrum with a Lorentzian line shape with a FWHM of $\Gamma = 2/\tau$.

c. Simulation of the time-resolved photoelectron spectrum

The TRPES at time Δt, $\sigma(E, \Delta t)$, was approximated as the Fourier transform of the wavepacket autocorrelation function corresponding to an initial state prepared by: (i) vertical excitation of the ground vibronic state to the $\pi\pi^*$ state at time $t = 0$, (ii) unitary time-evolution until time $t = \Delta t$, and (iii) vertical ionization to the cation manifold. That is,

$$\begin{aligned}\sigma(E, \Delta t) &\sim \int_{-\infty}^{\infty} \langle \Phi_0 | \hat{\epsilon}_1^{\dagger} e^{i\hat{H}\Delta t} \hat{\epsilon}_2^{\dagger} e^{-i\hat{H}t'} \hat{\epsilon}_2 e^{-i\hat{H}\Delta t} \hat{\epsilon}_1 | \Phi_0 \rangle e^{iEt'} dt' \\ &= 2Re \int_{0}^{\infty} \langle \Phi_0 | \hat{\epsilon}_1^{\dagger} e^{i\hat{H}\Delta t} \hat{\epsilon}_2^{\dagger} e^{-i\hat{H}t'} \hat{\epsilon}_2 e^{-i\hat{H}\Delta t} \hat{\epsilon}_1 | \Phi_0 \rangle e^{iEt'} dt',\end{aligned} \qquad \text{(Suppl. Eq. 18)}$$

where $|\Phi_0\rangle$ is the ground vibronic state, and $\hat{\epsilon}_1$ and $\hat{\epsilon}_2$ denote, respectively, vertical excitation and vertical ionization operators. $\hat{\epsilon}_1$ describes the excitation of the ground electronic state to the optically bright $|\pi\pi^*\rangle$ state. Let these be denoted by $|I^{(n)}\rangle$ and $|F^{(n)}\rangle$, respectively, where I and F index the "initial" and "final" neutral electronic states involved in the pump step, then

$$\hat{\epsilon}_1 = f_{FI} |F^{(n)}\rangle \langle I^{(n)}|, \qquad \text{(Suppl. Eq. 19)}$$

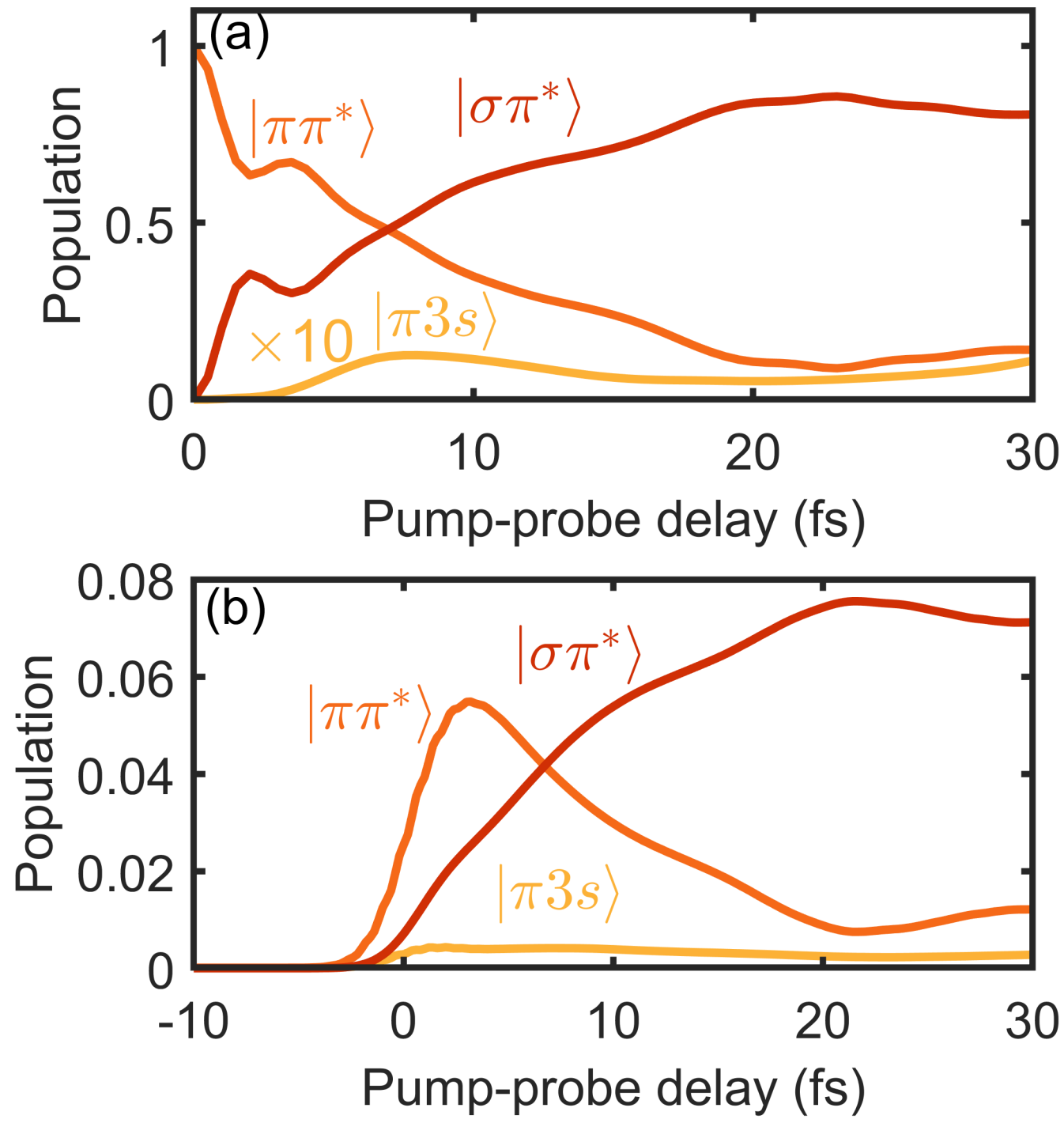

Suppl. Fig. 4: Diabatic electronic state population dynamics resulting from MCTDH calculations in ethylene at 158 nm using vertical excitation (a) and a 5 fs laser pulse (b). The calculation indicates that the population transfer to the $\pi 3s$ state remains negligible for both cases.

where f_{FI} is the corresponding oscillator strength, which we take within the Condon approximation. The operator $\hat{\epsilon}_2$ describes ionization from the neutral state manifold $\{|i^{(n)}\rangle\}$ to the cation state manifold $\{|i^{(c)}\rangle\}$, and is approximated as

$$\hat{\epsilon}_2 = \sum_{i,j} g_{ij}(\phi)|i^{(c)}\rangle\langle j^{(n)}|, \qquad \text{(Suppl. Eq. 20)}$$

where $g_{ij}(\phi)$ denotes the norms of the Dyson orbitals $\varphi_{ij}(r)$ corresponding to the ionization channel $|j^{(n)}\rangle \to |i^{(c)}\rangle$ taken as a function of the torsional angle ϕ. The Dyson orbitals are defined as

$$\varphi_{ij}(r) = \int dr_1 \cdots dr_{N-1} \psi_i^{(c)}(r_1, \ldots, r_{N-1}; \phi) \psi_j^{(n)}(r_1, \ldots, r_{N-1}, r_N; \phi), \tag{Suppl. Eq. 21}$$

where the $\psi_i^{(c/n)}$ are the wavefunctions associated with the electronic states $|i^{(c/n)}\rangle$. The squared norms of the Dyson orbitals are proportional to the corresponding total (photoelectron kinetic energy averaged) photionization cross-sections. As large amplitude nuclear motion occurs only along the torsional angle ϕ during the early time dynamics following excitation to the $|\pi\pi^*\rangle$ state, only the dependence of the Dyson orbital norms on this single nuclear degree of freedom is accounted for. This was achieved by expanding these terms in a $\cos^2(n\phi)$ basis:

$$g_{ij}(\phi) \approx g_{ij}(\phi_0) + \sum_{n=1}^{10} c^{(ij)} \cos^2(n\phi), \tag{Suppl. Eq. 22}$$

where ϕ_0 denotes the Franck-Condon point value of ϕ.

To minimize numerical artifacts associated with the use of a finite wavepacket propagation time T, the integrand in Equation Suppl. Eq. 18 was multiplied by the window function $G(t)$ (Equation Suppl. Eq. 16) and the damping function $F(t)$ (Equation Suppl. Eq. 17). Damping times τ of 50 and 30 fs were used for the $|\pi 3s\rangle$ and $|\pi\pi^*\rangle$ components of the spectrum.

We note that Equation Suppl. Eq. 18 corresponds to a description of the TRPES using first-order perturbation theory and the approximation of the pump and probe pulses as δ-functions, as originally derived and described in detail by Richings and Worth [14].

d. Parameterization of the model Hamiltonian and vertical ionization operator

The neutral electronic state basis $\{|i^{(n)}\rangle\}$ was taken to be comprised of the ten diabatic states corresponding to the ground state $|gs\rangle$ and the excited states $|\pi 3s\rangle$, $|\pi 3p_y\rangle$, $|\pi 3p_z\rangle$, $|\pi\pi^*\rangle$, $|\sigma\pi^*\rangle$, $|\pi 3p_x\rangle$, $|\pi 3d_{yz}\rangle$, $|\pi 3d_{z^2}\rangle$, and $|\sigma 3s\rangle$. The cation electronic state basis $\{|i^{(c)}\rangle\}$ was taken as the four states $|\pi^1\rangle$, $|\sigma_1^1\rangle$, $|\sigma_2^1\rangle$, $|\sigma_3^1\rangle$.

The expansion coefficients in the expansions of the neutral block $\boldsymbol{W}^{(n)}(\bar{\boldsymbol{Q}}, \phi)$ of the diabatic potential matrix (Equations Suppl. Eq. 11 and Suppl. Eq. 12) and the vertical ionization operator (Equation Suppl. Eq. 22) were determined by direct fitting to the quasi-diabatic potentials and Dyson orbital norms computed at a large number of displacements along each of the non-a_u normal modes $\bar{Q}_\alpha$ and the torsional angle ϕ. These were computed using the recently introduced quasi-diabatic variant of the second-order perturbative approximation of the combined density functional theory and multireference configuration interaction [QD-DFT/MRCI(2)] method [15]. In these calculations, the QE8 DFT/MRCI Hamiltonian was used [16], which uses the QTP17 exchange-correlation functional [17]. The aug-cc-pVTZ basis was used, along with the density fitting approximation in the evaluation of the two-electron integrals, for which the J&K aug-cc-pVTZ auxiliary basis set [18] was employed. The normal modes $\bar{Q}_\alpha$ and frequencies ω_α were computed at the B3LYP/aug-cc-pVTZ level of theory using the Turbomole set of programs [19]. The QD-DFT/MRCI(2) Franck-Condon point vertical excitation energies are given in Suppl. Table 4. Figure Suppl. Fig. 5 presents both *ab initio* data points as well as the fit potentials that arise from this procedure.

For the cation block $\boldsymbol{W}^{(c)}(\bar{\boldsymbol{Q}}, \phi)$ of the diabatic potential matrix, we made use of the pre-existing quadratic vibronic coupling Hamiltonian of Hazra and Nooijen [20], which provides an excellent description of the photoelectron spectrum of ethylene across the energy range of interest. To maximize agreement between the simulated and

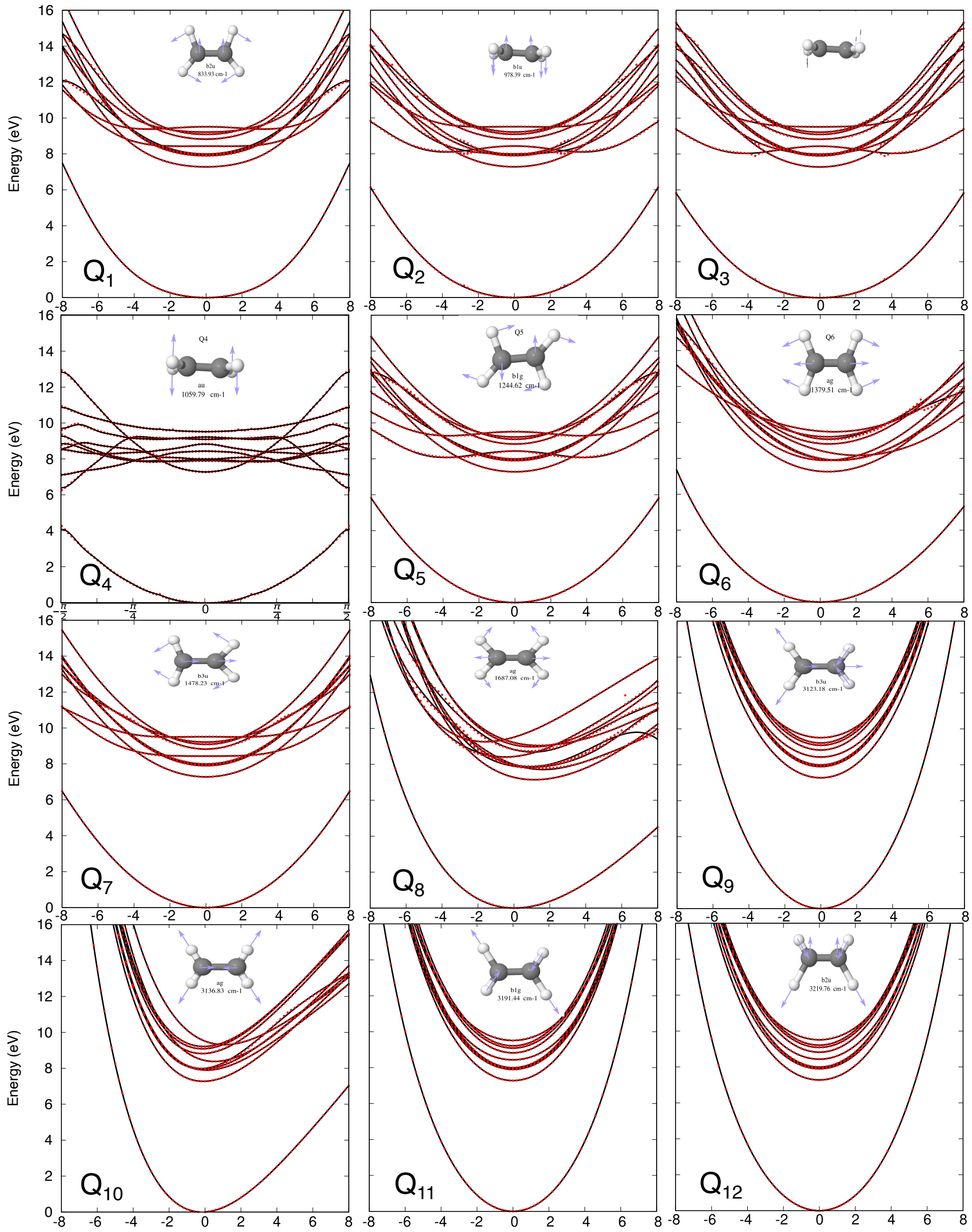

Suppl. Fig. 5: The QD-DFT/MRCI(2) explicitly computed diabatic states (points) and the fit potential curves (lines) employed in the vibronic Hamiltonian. Note that mode $\mathbf{Q}_4$ is a curvilinear dihedral coordinate.

experimental TRPES, the vertical ionization energies were slightly adjusted (see Suppl. Table 5). The model of Hazra and Nooijen is constructed in terms of the ground state normal modes, and so was modified via the replacement of the terms involving the a_u

CH_2 twisting mode with the trigonometric expansions in terms of the torsional angle ϕ given in Equations Suppl. Eq. 11 and Suppl. Eq. 12. The corresponding expansion coefficients were fitted to the results of QD-DFT/MRCI(2) calculations, entirely analogous to the construction of the neutral state block of the diabatic potential, with the same DFT/MRCI Hamiltonian, basis, and auxiliary basis sets being used.

The expansion coefficients entering into the model Dyson orbital norm surfaces $g_{ij}(\phi)$ (Equation Suppl. Eq. 22), were computed via least squares fitting to quasi-diabatic Dyson orbital norms computed along the torsional angle ϕ computed using the first-order corrected quasi-diabatic neutral and cation wavefunctions computed at the QD-DFT/MRCI(2) level of theory, again using the aug-cc-pVTZ basis. We refer the reader to Reference 15 for the definition and details of the computation of these quasi-diabatic wavefunctions.

The QD-DFT/MRCI(2)/aug-cc-pVTZ vertical excitation energies of the nine neutral excited states and four cation electronic states included in the model Hamiltonian are given in Suppl. Table 4 and Suppl. Table 5, respectively, alongside benchmark-level reference values.

e. Simulation of the interaction of the ground state with the pump pulse

The perturbative approach to the simulation of the TRPES discussed in Section c. Simulation of the time-resolved photoelectron spectrum requires that the interaction with the pump pulse be approximated by vertical excitation. In order to confirm the validity of this approximation, an additional wavepacket propagation calculation was performed in which the interaction of the ground vibronic state with the pump laser pulse was included. In these simulations, the following electric field-augmented Hamiltonian was used:

$$\hat{H}_{ML}(t) = \hat{H} - \sum_{i^{(n)},j^{(n)}} \boldsymbol{M}_{i^{(n)}j^{(n)}} \cdot \boldsymbol{E}(t)|i^{(n)}\rangle\langle j^{(n)}|. \qquad \text{(Suppl. Eq. 23)}$$

Here, $\boldsymbol{M}$ is the matrix representation of the dipole operator in the neutral state basis $\{|i^{(n)}\rangle\}$, and $\boldsymbol{E}(t)$ is the external optical electrical field. The latter was modeled as a normalized Gaussian envelope multiplying a cosine carrier wave,

$$\boldsymbol{E}(t) = \boldsymbol{e}\frac{S}{\sigma}\sqrt{\frac{4\ln 2}{\pi}} \exp\left(-\frac{4\ln 2}{\sigma^2}(t-t_0)^2\right)\cos(\omega(t-t_0)), \qquad \text{(Suppl. Eq. 24)}$$

where $\boldsymbol{e}$ denotes the polarization vector, σ is the FWHM of the pulse, ω is the central frequency, t_0 is the centre of the pulse, and S is a strength parameter used to set the peak intensity. The dipole matrix $\boldsymbol{M}$ was taken within the Condon approximation. That is, its nuclear coordinate-dependence was neglected. We note that this should be a non-too-severe approximation within a diabatic representation, as is used here. The pulse parameters were selected to be consistent with the experimental setup, with values of $\sigma = 5$ fs and $\omega = 158$ nm being used. The strength parameter S was set to 1.0.

Diabatic state populations for an isotropic distribution of molecular orientations were computed by numerically averaging the results over the laser polarization vector $\boldsymbol{e}$ using a 26-point Lebedev quadrature scheme [21, 22].

f. Wavepacket propagations

The wavepacket propagations required to simulate the VUV absorption and TRPES spectra were performed using the multi-layer multiconfigurational time-dependent Hartree (ML-MCTDH) method. The same matrix product states (MPS) type tree topology as described in Ref. 15 was used. In these calculations, harmonic oscillator

discrete variable representation (DVR) primitive basis functions were used for all normal modes $\bar{Q}_\alpha$, whilst an exponential DVR was used for the torsional angle ϕ, with a coordinate range of $\phi \in [-\pi, \pi)$. The ML-MCTDH tree is shown in Figure Suppl. Fig. 6. The numbers on each branch give the number of single-particle functions, with the exception of the bottom/leaf nodes, where the number of primitive basis functions are instead given. For reference, the symmetry, frequency, and a description of each normal mode are given in Suppl. Table 3. For the calculation of the ground state VUV absorption spectrum, a propagation time of 120 fs was used, yielding the wavepacket autocorrelation function up to 240 fs [23]. For the simulation of the TRPES, wavepacket propagations were performed for 100 fs following ionization to the cation manifold.

Mode	Symmetry	Frequency (cm^{-1})	Description
Q_1	b_{2u}	834	H-C-H in-plane rocking
Q_2	b_{1u}	978	H-C-H out-of-plane wagging
Q_3	b_{2g}	984	H-C-H out-of-plane wagging
Q_4	a_u	1060	H-C-H out-of-plane twisting
Q_5	b_{1g}	1245	C=C-H in-plane rocking
Q_6	a_g	1380	H-C-H in-plane scissoring
Q_7	b_{3u}	1478	H-C-H in-plane scissoring
Q_8	a_g	1687	C=C stretching
Q_9	b_{3u}	3123	C-H symmetric stretching
Q_{10}	a_g	3137	C-H symmetric stretching
Q_{11}	b_{1g}	3191	C-H asymmetric stretching
Q_{12}	b_{2u}	3220	C-H asymmetric stretching

Suppl. Table-3: Normal modes of ethylene computed at the B3LYP/aug-cc-pVTZ level of theory.

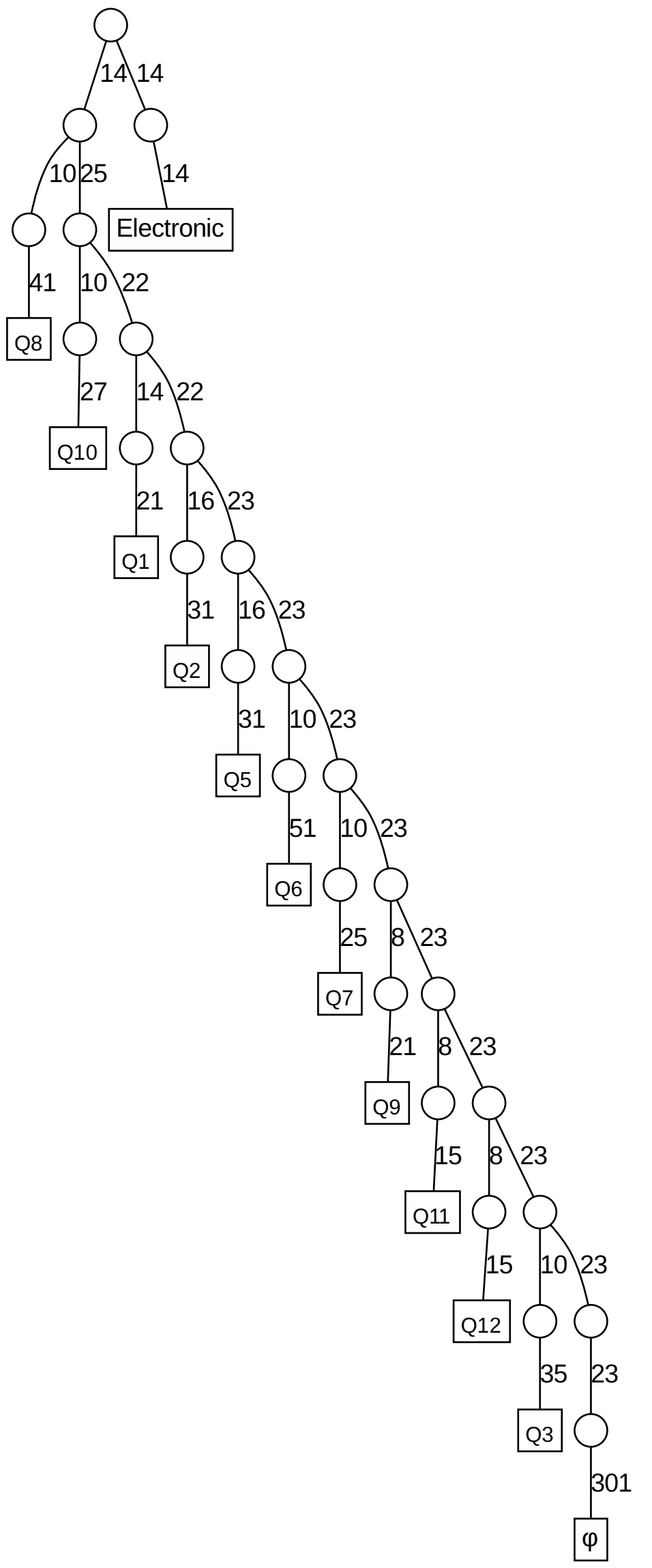

Suppl. Fig. 6: ML-MCTDH tree used in the calculation of the ground state VUV absorption spectrum and the TRPES. The numbers on each branch give the number of single-particle functions, except for the bottom/leaf nodes, where the number of primitive basis functions are instead given. For all normal modes Q_α, a harmonic oscillator DVR was used. For the torsional angle ϕ, an exponential DVR was used.

Supplementary Note 5: One-mode reduced densities

To analyze the role of the $|\sigma\pi^*\rangle$ state in driving torsional motion following excitation to the $|\pi\pi^*\rangle$ state, one-mode reduced densities for this degree of freedom were computed from the results of the ML-MCTDH wavepacket propagations. The total one-mode reduced density for the torsional angle ϕ is defined as

$$\rho(\phi, t) = \int \Psi^*(\phi, \bar{\boldsymbol{Q}}, t)\Psi(\phi, \bar{\boldsymbol{Q}}, t)d\bar{\boldsymbol{Q}}, \qquad \text{(Suppl. Eq. 25)}$$

where $\Psi(\phi, \bar{\boldsymbol{Q}}, t)$ is the total wavefunction associated with the state $|\Psi\rangle$. Furthermore, the one-mode reduced densities for the component of the wavepacket in just the $|\pi\pi^*\rangle$ and $|\sigma\pi^*\rangle$ states were computed. These state-specific reduced densities are defined as

$$\rho^{(i)}(\phi, t) = \int \Psi_i^*(\phi, \bar{\boldsymbol{Q}}, t)\Psi_i(\phi, \bar{\boldsymbol{Q}}, t)d\bar{\boldsymbol{Q}}, \qquad \text{(Suppl. Eq. 26)}$$

where $\Psi_i(\phi, \bar{\boldsymbol{Q}}, t)$ denotes the projection of the wavefunction onto the state $|i\rangle$.

The total one-mode reduced density computed following vertical excitation to the $|\pi\pi^*\rangle$ state is shown in Suppl. Fig. 7(a). The state specific one-mode reduced densities for the $|\pi\pi^*\rangle$ and $|\sigma\pi^*\rangle$ states are shown in Suppl. Fig. 7(b) and Suppl. Fig. 7(c), respectively. As can clearly be seen, the majority of the large amplitude torsional motion occurs in the $|\sigma\pi^*\rangle$ state. In other words, the vibronic coupling of the $|\pi\pi^*\rangle$ and $|\sigma\pi^*\rangle$ states is what drives the large-amplitude torsional motion.

Supplementary Note 6: Analysis of the ground state VUV absorption spectrum

The absorption spectrum $I(E)$ including the contributions from the $|\pi 3s\rangle$ and $|\pi\pi^*\rangle$ states is shown in Suppl. Fig. 8. Shown alongside is the experimental spectrum of Wu *et al.* [24]. Overall, good agreement between the two is found, with all main features of the spectrum being reproduced by the model. Namely, the broad, featureless background

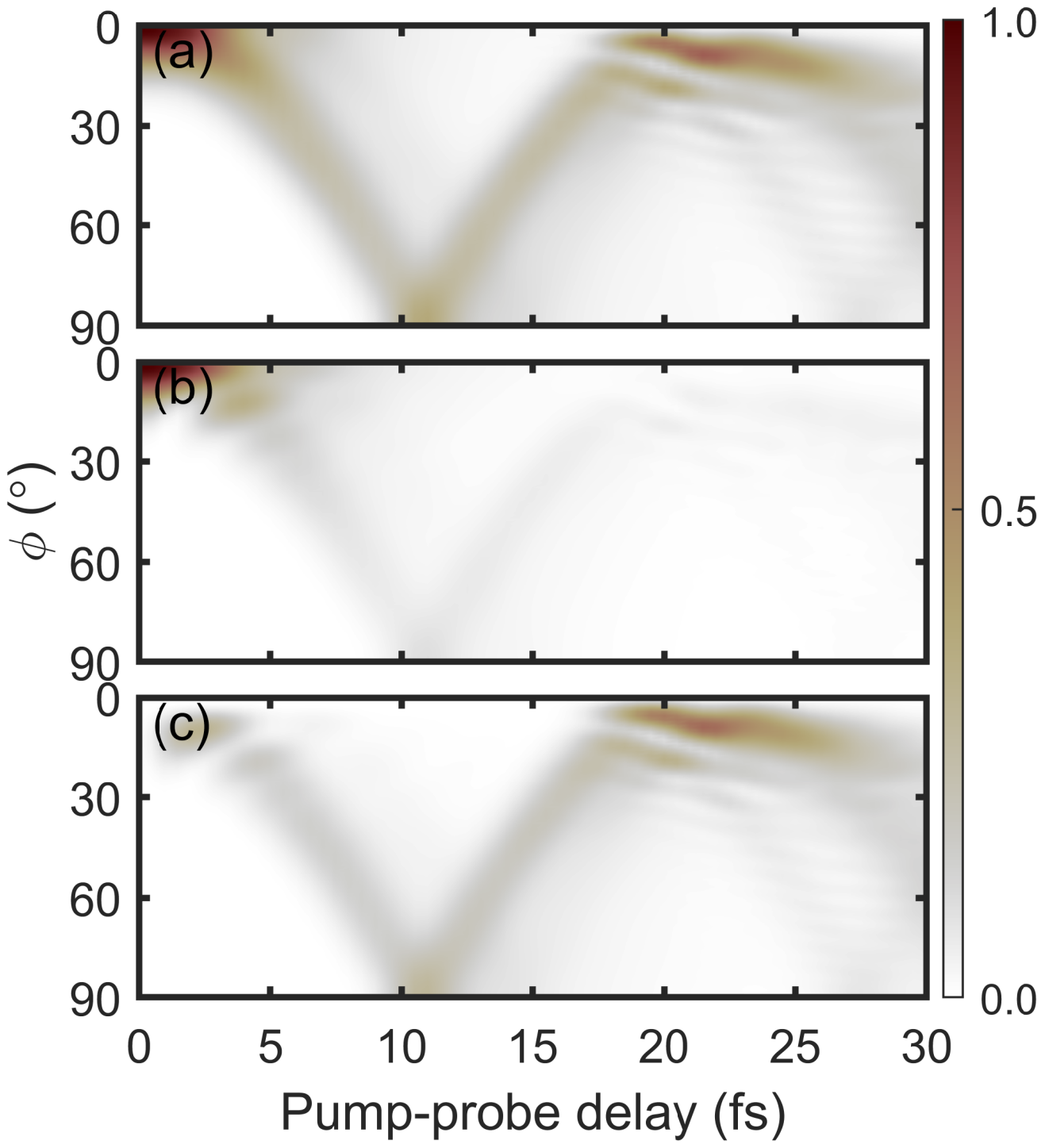

Suppl. Fig. 7: One-mode reduced densities for the torsional angle ϕ following vertical excitation to the $|\pi\pi^*\rangle$ state. (a) Total reduced density. (b) Reduced density for the $|\pi\pi^*\rangle$ component of the wavepacket. (c) Reduced density for the $|\sigma\pi^*\rangle$ component of the wavepacket.

from the $|\pi\pi^*\rangle$ state contribution, along with the progression of doublets corresponding to n quanta in the symmetric C=C stretching mode and either zero or two quanta in the non-totally-symmetric torsional mode.

To demonstrate the effect of the diabatic coupling between the $|\pi\pi^*\rangle$ and $|\sigma\pi^*\rangle$ states in the absorption spectrum, the $|\pi\pi^*\rangle$ contribution to it was recomputed with the corresponding off-diagonal block of the Hamiltonian zeroed. The result, shown in Suppl. Fig. 9, is striking, with the broad, featureless background now turned into a simple, well-resolved vibrational progression in the C=C stretching mode. From this, compelling evidence of the strong coupling of the $|\pi\pi^*\rangle$ and $|\sigma\pi^*\rangle$ states is provided.

State	Symmetry	Vertical Excitation Energy (eV)
$\|\pi 3s\rangle$	B_{1u}	7.28
$\|\pi 3p_y\rangle$	B_{2g}	7.90
$\|\pi 3p_z\rangle$	B_{3g}	7.97
$\|\pi\pi^*\rangle$	B_{2u}	7.99
$\|\sigma\pi^*\rangle$	B_{2g}	8.42
$\|\pi 3p_x\rangle$	A_g	8.82
$\|\pi 3d_{yz}\rangle$	A_u	9.09
$\|\pi 3d_{z^2}\rangle$	B_{1u}	9.21
$\|\sigma 3s\rangle$	B_{1g}	9.51

Suppl. Table-4: Vertical excitation energies of the neutral states of ethylene at the Franck-Condon point used in the model Hamiltonian. All values were computed at the QD-DFT/MRCI(2) level of theory using the aug-cc-pVTZ basis and QE8 Hamiltonian.

States	Symmetry	Vertical Ionization Energy (eV)
$\|\pi^1\rangle$	B_{3u}	10.61 (10.61)
$\|\sigma_1^1\rangle$	B_{3g}	13.06 (12.70)
$\|\sigma_2^1\rangle$	A_g	14.88 (14.88)
$\|\sigma_3^1\rangle$	$1B_{2u}$	16.25 (15.70)

Suppl. Table-5: Vertical ionization energies of the first four cation states of ethylene at the Franck-Condon point, computed at the IP-EOM-CCSD method and the TZ2P basis, and taken from Ref. 20. To attain maximal agreement between the simulated and experimental TRPES, these values were adjusted slightly. The values in parentheses are those used in the model Hamiltonian.

Supplementary Note 7: Validation of the vertical excitation approximation

The simulation of the TRPES employs the perturbative approach of Richings and Worth [14], in which the interaction of the ground state with the pump laser pulse

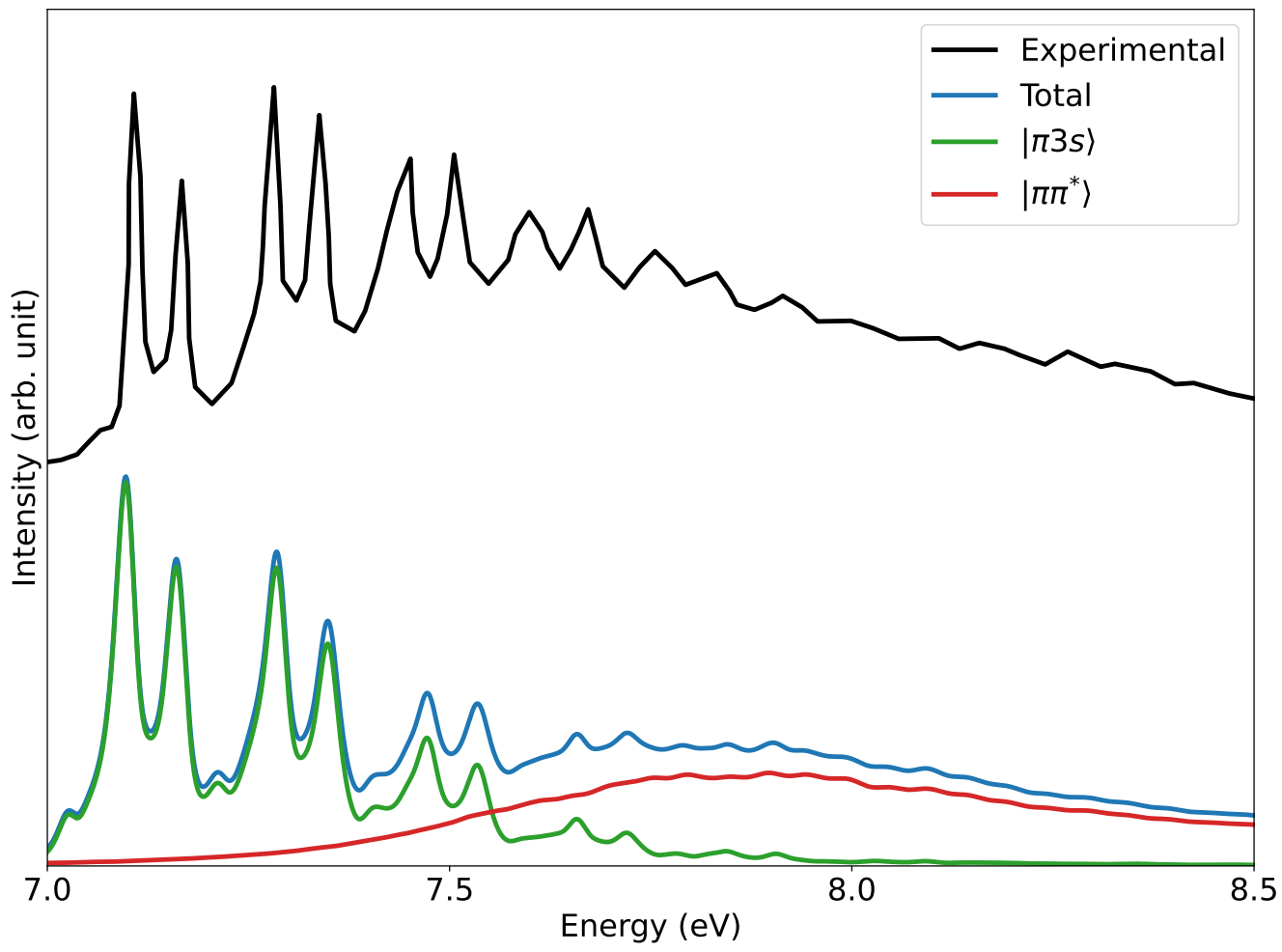

Suppl. Fig. 8: Computed absorption spectra of ethylene. The green line represents the contribution from the $|\pi 3s\rangle$ state, and the red line is the contribution from the $|\pi\pi^*\rangle$ state. The blue line shows the total simulated spectrum, computed as the sum of the two contributions. For comparison, the black line depicts the experimental absorption spectra reported by Wu *et al.* [24].

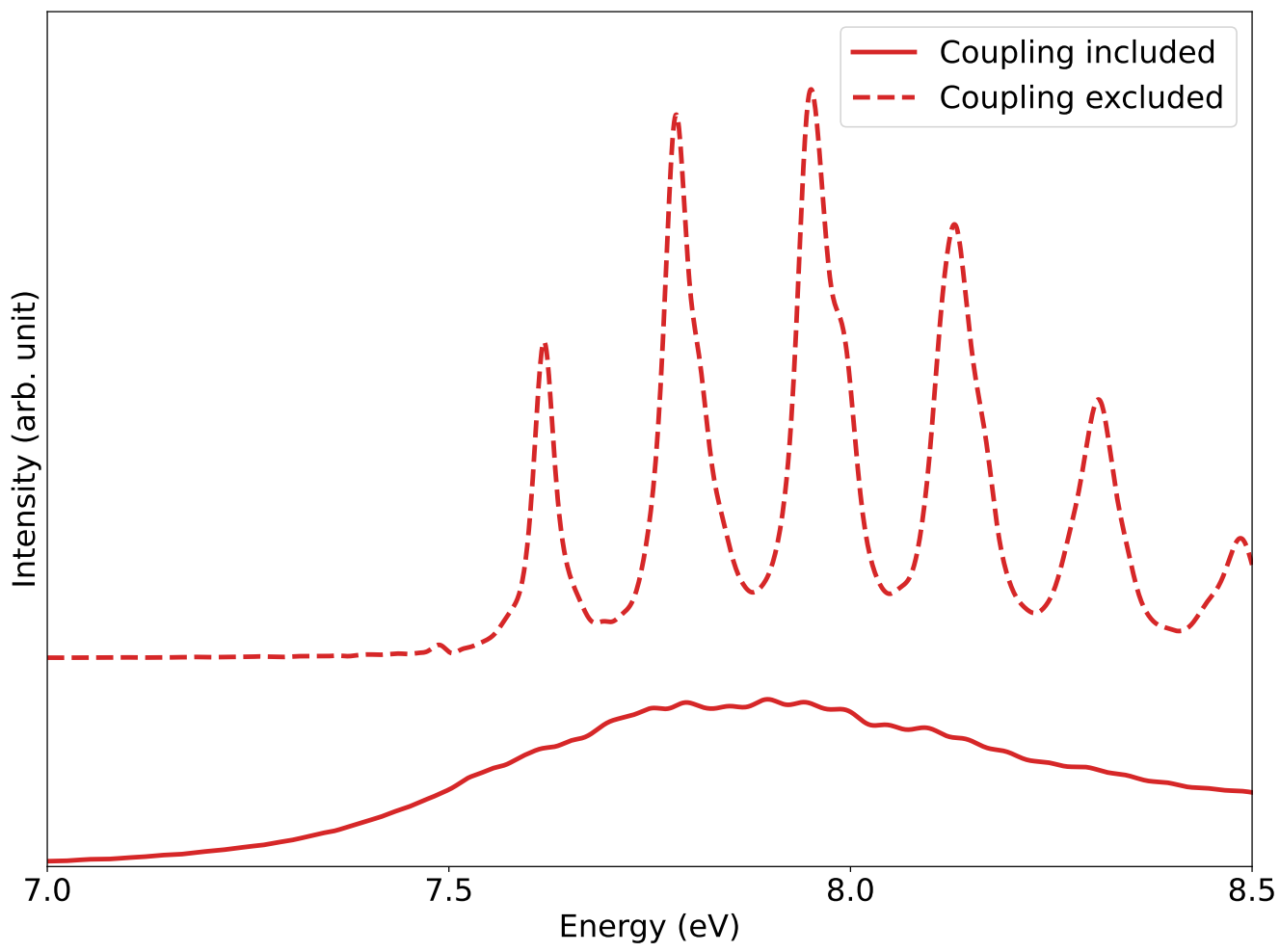

Suppl. Fig. 9: Comparison of the $|\pi\pi^*\rangle$ component of the absorption spectrum of ethylene computed with (solid line) and without (dashed line) the coupling to the $|\sigma\pi^*\rangle$ state included.

is replaced with a vertical excitation process to create the initial state. The severity of this approximation is expected to decrease with decreasing pump pulse duration,

and for the sub-5 fs duration of the pump pulse used in the present study, it seems well justified. To confirm this, the vertical excitation approximation was checked via comparison to the results of ML-MCTDH calculations including the interaction of the ground vibronic state with the pump laser pulse, as discussed in Section e. Simulation of the interaction of the ground state with the pump pulse. The resulting diabatic state populations are shown in Suppl. Fig. 4 alongside those computed following vertical excitation. In these calculations, the 158 nm pump laser pulse was centered at time $t_0 = 0$ fs. There are three main points to note. First, only the $|\pi\pi^*\rangle$ state is appreciably directly populated by the pump pulse. Second, the timescale for the transfer of population to the $|\sigma\pi^*\rangle$ state is very similar in both cases, with the maximum occurring at around 20 fs in each case. Third, no other state is appreciably populated within the first 30 fs. In each case, the $|\pi 3s\rangle$ state does become populated, but its population is essentially negligible compared to that of the $|\pi\sigma^*\rangle$ state. Considering these three areas of agreement, the vertical excitation approximation seems well justified overall.

Supplementary Note 8: Minimal diabatic state model

We discuss in more detail the minimal diabatic state model used to understand the early-time torsional dynamics following $\pi\pi^*$ excitation in ethylene. The two-state diabatic model, comprising the $|\pi\pi^*\rangle$ and $|\sigma\pi^*\rangle$ states, is informed by the results of full ten-state calculations of the dynamics following excitation to the $|\pi\pi^*\rangle$ state. As shown in Suppl. Fig. 4, only the $|\sigma\pi^*\rangle$ and $|\pi 3s\rangle$ states are populated within the first 30 fs. The $|\pi 3s\rangle$ population is essentially negligible, however, throughout this time interval. As such, it is possible to reduce the model Hamiltonian to a two-state sub-block, within the space spanned by the $|\pi\pi^*\rangle$ and $|\sigma\pi^*\rangle$ states alone. Such a two-state diabatic state model gives an excellent description of the initial torsional motion within the first few tens of femtoseconds following $\pi\pi^*$ excitation and, additionally, sheds new light on the origin of these dynamics.

In this minimal, two-state model, the potential matrix in the diabatic basis reads

$$\boldsymbol{W} = \begin{bmatrix} \langle \pi\pi^* | \hat{H}_{el} | \pi\pi^* \rangle & \langle \pi\pi^* | \hat{H}_{el} | \sigma\pi^* \rangle \\ \langle \sigma\pi^* | \hat{H}_{el} | \pi\pi^* \rangle & \langle \sigma\pi^* | \hat{H}_{el} | \sigma\pi^* \rangle \end{bmatrix}, \qquad \text{(Suppl. Eq. 27)}$$

where the on-diagonal elements correspond to the $|\pi\pi^*\rangle$ and $|\sigma\pi^*\rangle$ diabatic potentials, and the off-diagonal elements to the diabatic coupling between the two states. These are shown as a function of the torsional angle ϕ in Suppl. Fig. 10(a) and (b). Two main things are of note here. Firstly, the diabatic potential for the $|\pi\pi^*\rangle$ state has a positive curvature at the planar Franck-Condon point ($\phi = 0$), while the diabatic potential for the $|\sigma\pi^*\rangle$ state has negative curvature. Secondly, the diabatic coupling between the $|\pi\pi^*\rangle$ and $|\sigma\pi^*\rangle$ states becomes very large at twisted geometries. This has a large effect on the form of the corresponding adiabatic potential energy surfaces. To see this, we note that within the two-state model, the adiabatic potential energy surfaces can be computed as the eigenvalues of the 2×2 diabatic potential matrix $\boldsymbol{W}$:

$$\begin{bmatrix} \langle \pi\pi^* | \hat{H}_{el} | \pi\pi^* \rangle & \langle \pi\pi^* | \hat{H}_{el} | \sigma\pi^* \rangle \\ \langle \sigma\pi^* | \hat{H}_{el} | \pi\pi^* \rangle & \langle \sigma\pi^* | \hat{H}_{el} | \sigma\pi^* \rangle \end{bmatrix} = \boldsymbol{U}^{\dagger} \begin{bmatrix} V_- & 0 \\ 0 & V_+ \end{bmatrix} \boldsymbol{U}, \qquad \text{(Suppl. Eq. 28)}$$

where $\boldsymbol{U}$ denotes the matrix of eigenvectors of the diabatic potential matrix. The model adiabatic potential energy surfaces, V_- and V_+, are shown as a function of the torsional angle in Suppl. Fig. 10(c). The diabatic coupling between the $|\pi\pi^*\rangle$ and $|\sigma\pi^*\rangle$ states is sufficiently strong that the lower adiabatic potential, V_-, has negative curvature at the planar Franck-Condon point. This underlies the conventional understanding of how torsional motion is driven following $\pi\pi^*$ excitation. What the two-state diabatic model provides, however, is a mechanism for the existence of this negative curvature itself. Namely, the large diabatic coupling of the $|\pi\pi^*\rangle$ and $|\sigma\pi^*\rangle$ states.

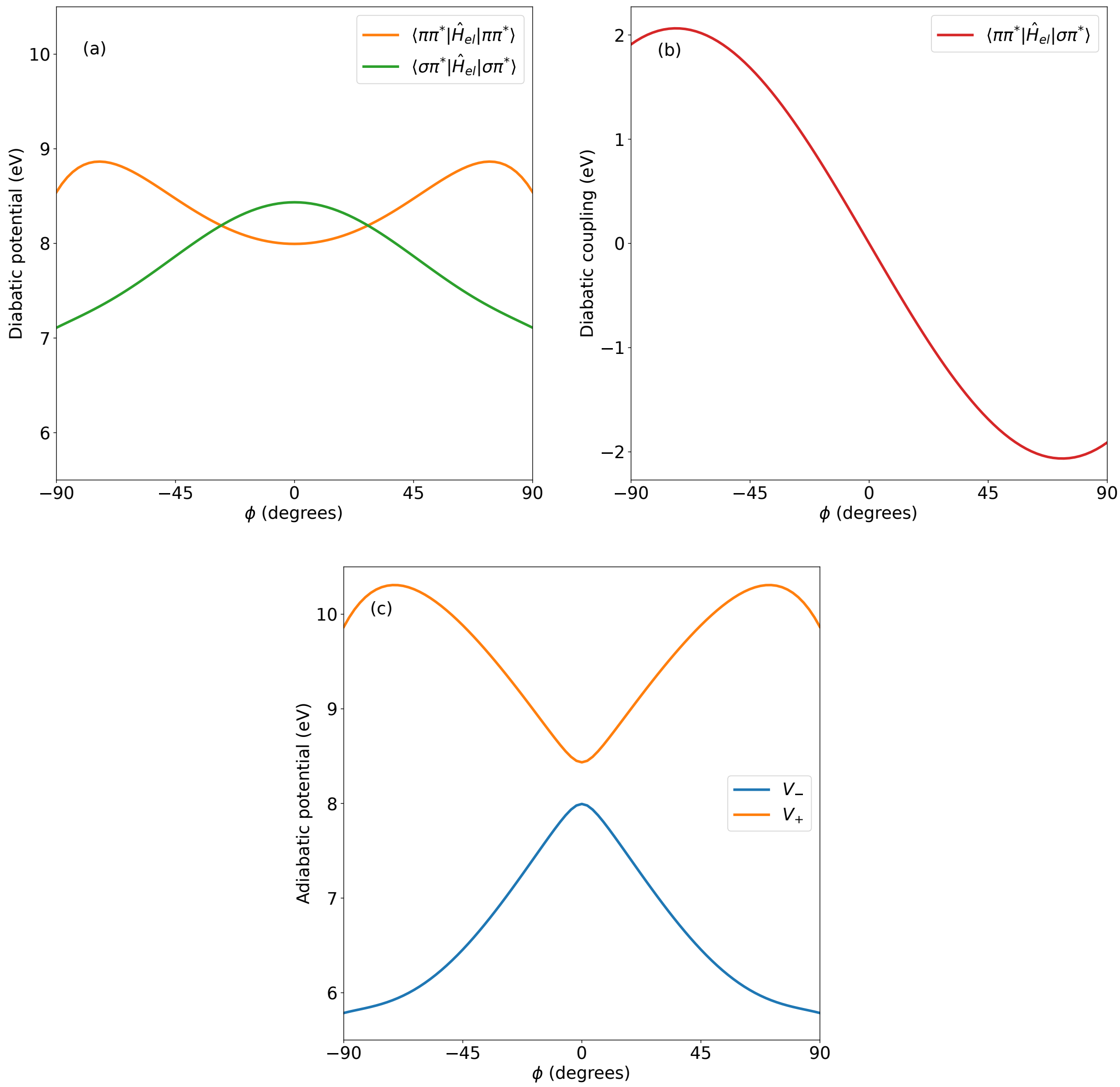

Suppl. Fig. 10: (a) $|\pi\pi^*\rangle$ and $|\sigma\pi^*\rangle$ diabatic potential energies as a function of the torsional angle ϕ. (b) Diabatic coupling between the $|\pi\pi^*\rangle$ and $|\sigma\pi^*\rangle$ states. (c) Model adiabatic potential energy surfaces resulting from the diagonalization of the two-state ($|\pi\pi^*\rangle$ and $|\sigma\pi^*\rangle$) diabatic potential matrix. The diabatic potentials and couplings were computed at the QD-DFT/MRCI(2) level of theory using the aug-cc-pVTZ basis.

Supplementary references

[1] Andrade, J. R. C. *et al.* Temporal characterization of tunable few-cycle vacuum ultraviolet pulses. *Nat. Photon.* **19**, 1240–1246 (2025).

[2] Kobayashi, T., Horio, T. & Suzuki, T. Ultrafast deactivation of the $\pi\pi^*$(V) state of ethylene studied using sub-20 fs time-resolved photoelectron imaging. *J. Phys. Chem. A* **119**, 9518–9523 (2015).

[3] Schepp, O., Baumann, A., Wieland, M., Azima, A. & Drescher, M. VUV-induced dynamics of the electronically excited C_2D_4 molecule in a single-color pump-probe experiment. *Chem. Phys. Lett.* **737**, 100024 (2019).

[4] Karashima, S., Humeniuk, A., Glover, W. J. & Suzuki, T. Ultrafast photoisomerization of ethylene studied using time-resolved Extreme Ultraviolet photoelectron spectroscopy. *J. Phys. Chem. A* **126**, 3873–3879 (2022).

[5] Boyer, A., Humeniuk, A., Karashima, S. & Suzuki, T. Deuterium isotope effect on internal conversion of ethylene studied by time-resolved photoelectron spectroscopy. *J. Phys. Chem. A* **128**, 7068–7072 (2024).

[6] Kosma, K., Trushin, S. A., Fuss, W. & Schmid, W. E. Ultrafast dynamics and coherent oscillations in ethylene and ethylene-d_4 excited at 162 nm. *J. Phys. Chem. A* **112**, 7514–7529 (2008).

[7] Tao, H. *et al.* Ultrafast internal conversion in ethylene. I. the excited state lifetime. *J. Chem. Phys.* **134**, 244306 (2011).

[8] Sension, R. J. & Hudson, B. S. Vacuum Ultraviolet resonance raman studies of the excited electronic states of ethylene. *J. Chem. Phys.* **90**, 1377–1389 (1989).

[9] Cattarius, C., Worth, G. A., Meyer, H.-D. & Cederbaum, L. S. All mode dynamics at the conical intersection of an octa-atomic molecule: Multi-configuration time-dependent Hartree (MCTDH) investigation on the butatriene cation. *J. Chem. Phys.* **115**, 2088–2100 (2001).

[10] Viel, A., Krawczyk, R. P., Manthe, U. & Domcke, W. Photoinduced dynamics of ethene in the N, V, and Z valence states: A six-dimensional nonadiabatic quantum dynamics investigation. *J. Chem. Phys.* **120**, 11000–11010 (2004).

[11] Köppel, H., Domcke, W. & Cederbaum, L. S. in *Multimode molecular dynamics beyond the born-oppenheimer approximation* 59–246 (John Wiley Sons, Ltd, 1984).

[12] Köppel, H., Domcke, W. & Cederbaum, L. S. in *The multi-mode vibronic-coupling approach* 323–367.

[13] Köppel, H., Domcke, W. & Cederbaum, L. S. Theory of vibronic coupling in linear molecules. *J. Chem. Phys.* **74**, 2945–2968 (1981).

[14] Richings, G. W. & Worth, G. A. The time-resolved photoelectron spectrum of toluene using a perturbation theory approach. *J. Chem. Phys.* **141**, 244115 (2014).

[15] Neville, S. P. & Schuurman, M. S. Calculation of quasi-diabatic states within the DFT/MRCI(2) framework: The QD-DFT/MRCI(2) method. *J. Chem. Phys.* **160**, 234109 (2024).

[16] Costain, T. S., Ogden, V., Neville, S. P. & Schuurman, M. S. A DFT/MRCI Hamiltonian parameterized using only *ab initio* data: I. valence excited states. *J. Chem. Phys.* **160**, 224106 (2024).

[17] Jin, Y. & Bartlett, R. J. Accurate computation of X-ray absorption spectra with ionization potential optimized global hybrid functional. *J. Chem. Phys.* **149**, 064111 (2018).

[18] Weigend, F. A fully direct RI-HF algorithm: Implementation, optimised auxiliary basis sets, demonstration of accuracy and efficiency. *Phys. Chem. Chem. Phys.* **4**, 4285–4291 (2002).

[19] TURBOMOLE. *TURBOMOLE V6.1* (2009). A development of University of Karlsruhe and Forschungszentrum Karlsruhe GmbH, 1989-2007, TURBOMOLE GmbH, since 2007. Available from http://www.turbomole.com.

[20] Hazra, A. & Nooijen, M. Vibronic coupling in the excited cationic states of ethylene: Simulation of the photoelectron spectrum between 12 and 18 eV. *J. Chem. Phys.* **122**, 204327 (2005).

[21] Values of the nodes and weights of ninth to seventeenth order gauss-markov quadrature formulae invariant under the octahedron group with inversion. *USSR Comput. Math. & Math. Phys.* **15**, 44 – 51 (1975).

[22] Quadratures on a sphere. *USSR Comput. Math. & Math. Phys.* **16**, 10 – 24 (1976).

[23] The calculation of autocorrelation functions for spectroscopy. *Chemical Physics Letters* **189**, 76–78 (1992).

[24] Wu, C. R., Chen, F. & Judge, D. Temperature-dependent photoabsorption cross sections in the VUV-UV region: Ethylene. *J. Geophys. Res.: Planets* **109** (2004).
