# Supplementary material for: Few-femtosecond photoelectron spectroscopy reveals fundamental photodynamics of the carbon-carbon double bond in ethylene
Source: Nat Commun. 2026 Jul 23;17:8991. doi: 10.1038/s41467-026-75782-3 (PMC13507108; doi:10.1038/s41467-026-75782-3)
Supplement: Supplementary file 2 [file 41467_2026_75782_MOESM2_ESM.pdf]

nature portfolio

Peer Review File

Few-Femtosecond Photoelectron Spectroscopy Reveals Fundamental Photodynamics of the Carbon–Carbon Double Bond in Ethylene

Corresponding Author: Dr Arnaud Rouzée

This file contains all reviewer reports in order by version, followed by all author rebuttals in order by version.

Version 0:

Reviewer comments:

Reviewer #1

(Remarks to the Author)
The manuscript by Arnab Sen et al reports a study of the isolated C=C double bond in photoexcited ethylene using sub-4-fs pulses centered at 158 nm and time-resolved photoelectron detection. The experimental results are discussed in terms of a minimal model Hamiltonian parametrized according to first principles calculations. Specifically, the authors show that the TRPES data can be well described by a reduced version of a vibronic coupling model Hamiltonian comprising the π-π* and σ-π* states coupled through the torsional mode. Neglecting this vibronic coupling is not sufficient to reproduce the experiment.

The topic is highly interesting given the role of conjugated π systems as building blocks of organic functional materials, such as those used in light harvesting, solar cells and molecular electronics, where C=C bonds are ubiquitous. Additionally, vibronic coupling involving C=C bonds is being recognized to play an key role in light-induced ultrafast dynamics in these materials, often dominating their photophysics and photochemistry. In this context, ethylene represents a benchmark problem for light-driven processes in larger functional materials. Experimental access to vibronic coupling of the π-π* and σ-π* state is essential for a complete and in-depth understanding of excited state dynamics. Therefore, I believe this will be of interest to the field.
However, in its present form, the manuscript presents some issues that need to be addressed before a decision on publication can be made. Below are my main suggestions:

1) To help the readers better follow the discussion, it would help providing some conceptual scheme, in particular for the discussion of the experimental results on page 5. The authors may consider merging Fig. 1 and 2, or refer more directly to the PES in Fig. 1 when discussing the photoelectron spectra of Fig. 2, or providing additional illustrations of the transitions directly in Fig. 2 to help the reader. Along the same lines, providing a scheme of the ethylene molecule with indication of the torsional mode, and adding a conceptual illustration of the experiment may be helpful for a broader (non-expert) readership.

2) Page 1, first line of the abstract: the statement that “conjugated C=C double bonds are the most important organic chromophores” is quite absolute. It would be helpful to clarify if they refer to isolated C=C bonds or conjugated π systems in general.

3) Please briefly explain the rationale behind the comparison with the deuterated molecule in the experiments and what is learnt from this comparison for the conclusions.

4) A brief discussion of previous recent work addressing the ultrafast dynamics of $C_2H_4^+$ cation (e.g., DOI: 10.1126/science.abf1656 ; https://doi.org/10.1021/acs.jpclett.2c02763; …) would be helpful. While those studies address ionized states, they probe similar nuclear coordinates and nonadiabatic relaxation. I believe it would help placing the present results in a broader context.

5) Page 3, lines 80-82: the sentence “Importantly, this new model is consistent with previously reported …” requires at least one reference to the mentioned previous trajectory-based dynamics simulations. Similarly on page 7, lines 145-146: the statement that the result “is consistent with many experimental observations” needs references when referring to previous

works.

6) At the end of the introduction, the authors write that “this new model … will have broad implications for studying and controlling light-induced energy conversion, … “. However, the manuscript at this stage does not suggest specific strategies or concrete examples in this direction. Could the authors elaborate on this?

7) The oscillations in Fig. 2 features (iii) and (iv) appear out of phase in both C2H4 and the deuterated counterpart. Can the authors comment more extensitvely on the origin? Please also comment more on the timescale of the rapid relaxation of these oscillations. In the simulated TRPES, it seems significantly faster damping. What is the origin of damping in the simulations?

8) The conclusions strongly rely on results from the reduced model Hamiltonian used to reproduce the experimental TRPES. While the model is detailed in the Supplementary, it would be helpful to discuss more directly in the manuscrtipt limitation, similarities and differences between this minimal diabatic model and established linear vibronic coupling frameworks.

9) In the conclusion paragraph (page 13), the authors mention combining experiments with ab initio quantum dynamics simulations. However, the main conclusions seem based primarily on the reduced model Hamiltonian, which is parametrized using ab initio results. I think they should better clarify the ab initio aspects and explain to which extent conclusions are based on ab initio calculations in comparison to parametrized model Hamiltonian. Moreover, on the same page 13, line 263, I would recommend to specify more clearly the “high-level quantum dynamics simulations” as it sounds somewhat vague.

10) Page 17, lines 364-368: this seems to be guideline or template text. Please check

11) Finally, I suggest to revise the title to reflect more directly the main message of the paper. This is, however, a matter of taste and thus optional and for the authors to decide

Reviewer #2

(Remarks to the Author)
The present manuscript reports a combined experimental and theoretical study of the dynamics induced upon photo-excitation of ethylene, a prototypical system for carbone-carbone double bond. The molecule is excited with a VUV pump pulse to the bright pi,pi* state. The induced dynamics is then probed with a VUV probe pulse through time-resolved photoelectron spectroscopy (TRPES), with a sub-4 fs time resolution. The better time resolution allows to observe new features in the TRPES. The experimental observations are accompanied by state-of-the-art quantum dynamics simulations using the MCTDH method. The TRPES is also simulated and shows a very good agreement with the measurements. Regarding the interpretation of the experimental features, the authors suggest to use a diabatic basis, rather than the adiabatic one. By doing so, the present work shows the implication of a dark sigma,pi* state that gets populated shortly after the excitation and that can explain some of the experimental features. Although this allows to propose a different mechanism for the process, it is noted that the choice of electronic basis does not change the simulated dynamics, nor TRPES results, and « only » provides an alternative interpretation of it.

(i) Following down this line, the choice of diabatic states is not unique and one could have chosen a different diabatic basis, leading to yet another alternative interpretation. From the shape of the adiabatic curves (Figure 1a), one could have, for instance, expected diabatic curves much closer to the adiabatic ones for large torsional angles, and which are energetically degenerate and « cross » around phi=0. Can the authors comment on this alternative choice of diabatic states? It may be that the diabatic states are chosen so that their energy should be equal to the adiabatic ones for a planar geometry, meaning a diabatic energy gap equal to the adiabatic one and a zero coupling at phi=0. This can be a choice but does not necessarily need to be that way. What diabatic states (and subsequent interpretation) would be obtained in case of a choice of a 50/50 mix at phi=0?

I also have some technical questions regarding the simulations:
(ii) Hamiltonian models are often obtained through a least-square fitting procedure against ab initio points, and comparisons of the fitted adiabatic models are typically shown to validate the model with respect to the ab initio points. Here, it is mentioned that the fitting procedure was performed directly in a quasi-diabatic basis. The authors should show the comparison between the fitted model and the quasi-diabatic potential points.
(iii) The out-of-plane twisting normal mode is replaced with a curvilinear torsional coordinate. Have the other normal modes been re-orthogonolised with respect to this torsional coordinate?

A detail:
(iv) Page 7: « the decease in vertical ionisation energy with increasing torsional angle ». Is it not the *increase* of ionisation energy?

Reviewer #3

(Remarks to the Author)
The work by Sen et al. is a joint experimental and theoretical work focusing on the excited state dynamics of ethylene, the archetype of cis-trans photoisomerization, after 160 nm excitation. The time-resolved photoelectron spectroscopy experiment

is enabled by sub-4 fs VUV pulses generated via RDW emission, that constitute both the pump and the probe of the (1+1) photoionization process.
RDW is an emerging technology in ultrafast science; still, published time-resolved experiments based on it are very limited. The work by Sen et al. showcases very convincingly the power of the method: I'm not aware of any other technique capable of delivering sub-4 fs pulses at 160 nm. The application to ethylene is very natural and compelling, in view of the fundamental importance of its dynamics.
Both the experimental traces in C_2H_4 and C_2D_4 exhibit extremely rich dynamics unfolding in the first tens of fs. This exceptional data quality enables very detailed comparison with advanced simulations, ultimately demonstrating that the 1B3g(σπ*) state is strongly coupled by torsional motion to the optically populated 1B1u(ππ*) state.
It's worth noting that none of the existing datasets on the same dynamics in literature (for instance refs. 24 and 25, which I personally considered to be the best available up to now) would have nearly allowed for a similarly detailed comparison with theory: the benefits of the improved temporal resolution are striking.
The conclusions reached by theory are very convincing, also in view of the remarkable agreement with the experimental data: it's indeed very interesting that the coupling between ππ* and σπ* states drives the torsional motion, and it's surprising that this was not previously recognized. I share the authors' view that this phenomenon is general, and it impacts our understanding of photoisomerization around a C=C double bond in several systems.
The manuscript is very well written.
For all the reasons above, I think the work could be suitable for publication in Nature Communications, if the following points are properly addressed:
1- Population transfer to the ground state
The experimental datasets extend to 120 fs delay; the simulations instead stop at 30 fs. While the limited temporal window is still sufficient to discuss the main point of the paper - the coupling between ππ* and σπ* states - I believe it would be very important to extend the simulations to longer delays to catch the ground state recovery and complete the story.
For instance, according to ref. 24, "The nuclear wave packet created by a 160 nm pump pulse accesses the C=C twisted geometries within 10 fs, and the population transfer from the excited to the ground state occurs within the next 20–30 fs"; the authors instead comment on p. 10 that no electronic state other than σπ* is significantly populated within the simulation time (and then corroborate this in Figure S3).
The rapidly decreasing kinetic energy in the experimental datasets still suggests that the ground state is starting to recover already within the first 100 fs; however, the probe energy limits the observation window and doesn't allow for a direct identification, as it's common for VUV-probe TRPES. Still, given the excellent agreement with theory in the first 30 fs, I believe that the simulations alone would be sufficient in determining the timing of ground state recovery.

2- Identification of ππ* and σπ* states via PADs
As the experiment is performed with a VMI, I believe it would be of interest to determine whether the available angular resolution can play a role in aiding the interpretation. Could the authors comment on the capability to distinguish ππ* - and σπ* -originated photoelectrons based on their angular distributions (PADs)?

Version 1:

Reviewer comments:

Reviewer #1

(Remarks to the Author)
The authors have addressed all questions and concerns in detail and revised the manuscript accordingly. I have no further comments and I would recommend the publication of the revised manuscript.

Reviewer #2

(Remarks to the Author)
The authors have addressed my comments. They have in particular included in the SI (Figure S4) the comparison of the Hamiltonian model against the ab initio electronic structure data. I note a discrepancy along normal mode 8, with the fitted model going down in energy for large positive Q values. Can the authors comment on this discrepancy and the potential impact it may have on the dynamics?

Reviewer #3

(Remarks to the Author)
The authors appropriately answered my comments.

1- It's indeed a pity that the vibronic Hamiltonian fails to describe relaxation to the ground state: I now fully understand the point. I thus suggest the authors to point this out in the main text, as the limitations of the employed model may not be straightforward to the non-specialized reader. For the same reason, I also suggest to mention the experimental limitations related to the observation window in following the relaxation pathway back to the ground state.

2- I appreciate the analysis of the asymmetry parameters and the related discussion provided by the authors. I suggest the authors to include it in the Supplementary Informations, as it's a very useful addition for the TRPES community.

In my opinion, the revised manuscript deserves publication in Nature Communications.

Open Access This Peer Review File is licensed under a Creative Commons Attribution 4.0 International License, which permits use, sharing, adaptation, distribution and reproduction in any medium or format, as long as you give appropriate credit to the original author(s) and the source, provide a link to the Creative Commons license, and indicate if changes were made.
In cases where reviewers are anonymous, credit should be given to 'Anonymous Referee' and the source.
The images or other third party material in this Peer Review File are included in the article's Creative Commons license, unless indicated otherwise in a credit line to the material. If material is not included in the article's Creative Commons license and your intended use is not permitted by statutory regulation or exceeds the permitted use, you will need to obtain permission directly from the copyright holder.
To view a copy of this license, visit https://creativecommons.org/licenses/by/4.0/

We thank all three reviewers for carefully reading our manuscript and their suggestions. We have performed several modifications of the manuscript according to the referees' comments, which we believe have significantly improved the manuscript. Our answers to the questions and comments are detailed in the point-by-point response below.

Reviewer 1:

The first referee acknowledges that the topic is highly relevant given the important role of π conjugated systems in numerous applications and that our study will be of interest to the field. The referee points out few issues that needed to be addressed.

1. To help the readers better follow the discussion, it would help providing some conceptual scheme, in particular for the discussion of the experimental results on page 5. The authors may consider merging Fig. 1 and 2, or refer more directly to the PES in Fig. 1 when discussing the photoelectron spectra of Fig. 2, or providing additional illustrations of the transitions directly in Fig. 2 to help the reader. Along the same lines, providing a scheme of the ethylene molecule with indication of the torsional mode, and adding a conceptual illustration of the experiment may be helpful for a broader (non-expert) readership.

We thank the referee for this useful comment. We have revised the first figure of the manuscript to include a simplified schematic of the experiment (Fig. 1a). We also merged Figs. 1 and 2 to facilitate the identification of the ionization channels based on the potential energy surfaces. In addition, in the inset of Fig. 1d, we added the assignment of the photoelectron bands observed at t=0 (i.e., at the equilibrium geometry), which are also indicated by arrows on the PESs in Fig. 1b.

2. Page 1, first line of the abstract: the statement that "conjugated C=C double bonds are the most important organic chromophores" is quite absolute. It would be helpful to clarify if they refer to isolated C=C bonds or conjugated π systems in general.

We agree with the referee that the sentence might be confusing. We have therefore reformulated the sentence to make it more general.

Conjugated π-systems, often composed of carbon–carbon double bonds as key structural units, are arguably among the most important organic chromophores and serve as the paradigm for $\pi\pi^*$ excitation.

3. Please briefly explain the rationale behind the comparison with the deuterated molecule in the experiments and what is learnt from this comparison for the conclusions.

In general, comparison between the non-deuterated and deuterated molecules provides a direct means to identify the vibrational modes involved in the dynamics, as their frequencies are expected to scale with the square root of the inverse mass. By analyzing how the observed oscillation frequencies change upon deuteration, one can therefore assign the dominant nuclear motions underlying the process. In our measurements, depending on the photoelectron kinetic energy, the extracted frequencies scale by a factor between 1.3 and 1.41, in good agreement with the $\sqrt{2}$ dependence expected for torsional motion. This supports the assignment of the observed oscillations to torsion about the C–C bond. A more detailed discussion is provided in the "Quantitative Analysis of TRPES" section of the Supporting Information. This point was further discussed in the article: "Similar behavior is observed in both C_2H_4 and C_2D_4, with a slight reduction in the initial decay rate and increases in the oscillation period for the latter. This is consistent with the expected changes of vibrational frequencies upon deuteration (see Supplementary Information and [1]). In

particular, the initial decay observed in feature (i) in C_2D_4 is slower by a factor of close to $\sqrt{2}$ compared to C_2H_4, consistent with the D/H mass ratio."

4. A brief discussion of previous recent work addressing the ultrafast dynamics of $C_2H_4^+$ cation (e.g., DOI: 10.1126/science.abf1656 ; https://doi.org/10.1021/acs.jpclett.2c02763; . . .) would be helpful. While those studies address ionized states, they probe similar nuclear coordinates and nonadiabatic relaxation. I believe it would help placing the present results in a broader context.

We thank the referee for this helpful suggestion. We agree that nonadiabatic relaxation also plays a critical role following ionization, as demonstrated in several recent studies of the $C_2H_4^+$ cation, including the works by K. S. Zinchenko *et al.* (DOI: 10.1126/science.abf1656) and M. Lucchini *et al.* (DOI: 10.1021/acs.jpclett.2c02763). While our manuscript focuses on nonadiabatic excitation dynamics in neutral ethylene in the VUV spectral range, these studies probe similar nuclear coordinates and highlight related ultrafast relaxation pathways. To better place our results in this broader context, we have added the following sentence to the manuscript:

These interactions also play a central role in the ultrafast electronic relaxation of the $C_2H_4^+$ cation, as recently observed [11,12].

5. Page 3, lines 80-82: the sentence "Importantly, this new model is consistent with previously reported . . . " requires at least one reference to the mentioned previous trajectory-based dynamics simulations. Similarly on page 7, lines 145-146: the statement that the result "is consistent with many experimental observations" needs references when referring to previous works.

We thank the referee for pointing this out. We agree that appropriate references are needed to support these statements. We have now added relevant citations on page 3 (lines 80–82) to support the comparison with previous trajectory-based dynamics simulations, and on page 7 (lines 145–146) to substantiate the consistency with prior experimental observations.

6. At the end of the introduction, the authors write that "this new model . . . will have broad implications for studying and controlling light-induced energy conversion, . . . ". However, the manuscript at this stage does not suggest specific strategies or concrete examples in this direction. Could the authors elaborate on this?

We thank the referee for this important comment. We agree that the original statement was somewhat general and could benefit from further clarification. In this context, our results suggest that modifying the molecular geometry, for instance through chemical substitution, can influence the coupling between the relevant electronic states, and thereby affect the nonadiabatic relaxation pathways and the outcome of the isomerization process. Such control over the electronic coupling provides a possible route to steer the photochemical dynamics. To reflect this in the manuscript, we have now added the following sentence at the end of the introduction:

Our results suggest that modifying the molecular geometry, for instance through chemical substitution, can influence the coupling between the relevant electronic states, and thereby affect the nonadiabatic relaxation pathways and therefore the outcome of the photochemical dynamics.

7. The oscillations in Fig. 2 features (iii) and (iv) appear out of phase in both C2H4 and the deuterated counterpart. Can the authors comment more extensitvely on the origin? Please also comment more on the timescale of the rapid relaxation of these oscillations. In the simulated TRPES, it seems significantly faster damping. What is the origin of damping in the simulations?

We thank the referee for these insightful comments.

Regarding the apparent out-of-phase oscillations observed in features (iii) and (iv), we believe that this behavior does not reflect a direct dynamical phase relation but rather arises from the distinct ionization channels contributing to these features. Based on our simulations, features (iii) and (iv) originate from the $\sigma\pi^* \rightarrow \pi^1$ and $\sigma\pi^* \rightarrow \sigma_3^1$ ionization channels, respectively. Importantly,

the latter channel is only energetically accessible at large torsional angles. This is illustrated in Fig. 1b, which shows the diabatic potential energy curves as a function of the torsional coordinate: at a torsional angle of 0°, the probe photon energy is insufficient to access the σ_3^1 ionic state. As a result, the two features probe different regions of the nuclear wavepacket along the torsional coordinate, which can give rise to an apparent phase difference without implying a strict out-of-phase vibrational motion.

Concerning the damping of the oscillations, the experimentally observed rapid decay is most likely due to population transfer out of the excited $\sigma\pi^*$ state via nonadiabatic transitions, particularly near a torsional angle of $\sim 90°$, where strong coupling to the ground state is expected. In contrast, the simulated TRPES exhibits significantly weaker damping because such coupling to the ground state is not fully captured in our model. In particular, the present description does not accurately account for nonadiabatic population transfer at large torsional angles, and therefore the $\sigma\pi^*$ population (Fig. 3) does not exhibit substantial decay (see comment 2 of third referee for further details).

8. The conclusions strongly rely on results from the reduced model Hamiltonian used to reproduce the experimental TRPES. While the model is detailed in the Supplementary, it would be helpful to discuss more directly in the manuscript limitation, similarities and differences between this minimal diabatic model and established linear vibronic coupling frameworks.

To clarify, the quantum dynamics calculations in both the neutral and cationic manifolds, as well as the simulations of the time-resolved photoelectron spectrum, were performed using a full-dimensional electronic-state description comprising 10 neutral and 4 cationic states. The reduced two-state representation introduced here is a simplified picture derived from an analysis of the full-dimensional model, intended to highlight the key physical insights emerging from the *ab initio* treatment. In particular, it emphasizes that the early-time dynamics of ethylene are primarily governed by strong vibronic coupling between the $|\pi\pi^*\rangle$ and $|\sigma\pi^*\rangle$ diabatic states.

We've amended the following text to the manuscript to make role of the different models more explicit:

In page 8, we replaced: "Here, we introduce a new minimal diabatic state model" by:
Here, we introduce a new minimal diabatic state representation.

In page 9, we replaced: " Accordingly, the system can therefore be described by a simple reduced, two-state model, consisting of the strongly vibronically coupled $|\pi\pi^*\rangle$ and $|\sigma\pi^*\rangle$ diabatic states." by:
The photo-physics of ethylene considered here can therefore be qualitatively described by a simple two-state representation, consisting of the strongly vibronically coupled $|\pi\pi^*\rangle$ and $|\sigma\pi^*\rangle$ diabatic states.

In page 10, we also replaced: "Validation of this new model, and the strong coupling between the $|\pi\pi^*\rangle$ and $|\sigma\pi^*\rangle$ states, is provided by its use in the simulation of the VUV absorption spectrum. As discussed in the Supplementary Information, the model Hamiltonian was used in conjunction with the multi-layer multicon-figurational time-dependent Hartree (ML-MCTDH) method [46–53] to simulate the absorption spectrum in the 6-8 eV range." by:
To validate this new interpretation and, critically, the previously unappreciated strong coupling between the $|\pi\pi^*\rangle$ and $|\sigma\pi^*\rangle$ states, the VUV absorption spectrum of ethylene in the 6-8 eV range was first simulated using the full-dimensional, ten-state model Hamiltonian in conjunction with the multi-layer multiconfigurational time-dependent Hartree (ML-MCTDH) method [46–53].

In page 10, the sentence: "We now turn to the diabatic state populations computed following vertical excitation to the $|\pi\pi^*\rangle$ state that is populated upon excitation at 158 nm." was modified by:
We now turn to the diabatic state populations computed using the full-dimensional, ten-state model Hamiltonian following vertical excitation to the $|\pi\pi^*\rangle$ state that is populated upon excitation at

158 nm.

In page 10, we also completed the sentence: "We note that no other electronic state is significantly populated within this time." by:
We note that no other electronic state is significantly populated within this time, strongly supporting that the early-stage dynamics in ethylene can be well described using a simplified diabatic two-state representation as discussed above.

Finally, we also added in the caption of Figs. 2 and 3 that simulations were performed using the full-dimensional, ten-state model Hamiltonian.

9. In the conclusion paragraph (page 13), the authors mention combining experiments with ab initio quantum dynamics simulations. However, the main conclusions seem based primarily on the reduced model Hamiltonian, which is parametrized using ab initio results. I think they should better clarify the ab initio aspects and explain to which extent conclusions are based on ab initio calculations in comparison to parametrized model Hamiltonian. Moreover, on the same page 13, line 263, I would recommend to specify more clearly the "high-level quantum dynamics simulations" as it sounds somewhat vague.

We thank the referee for this important comment. We would like to clarify again here that all simulations presented in the manuscript, in particular those shown in Figs. 3 and 4 (now Figs. 2 and 3), were performed using the full model Hamiltonian. This model is constructed from *ab initio* electronic structure calculations and includes up to 10 coupled electronic states, without any empirical adjustment of parameters.

The reduced two-state model introduced in the manuscript is derived directly from this full *ab initio*-based description and is used solely to provide a simplified and intuitive interpretation of the underlying dynamics. The main conclusions of the work are therefore supported by the full-dimensional simulations, while the reduced model serves to rationalize the key physical mechanisms.

We agree with the referee that this distinction was not sufficiently clear in the original manuscript. We have therefore revised the caption to explicitly state which model is used in Figs. 3 and 4, and the text accordingly (see answer to comment 8). In addition, we have specified more clearly what is meant by "high-level quantum dynamics simulations" in the conclusion and added the following

Finally, our work highlights the importance of performing experiments at extremely high temporal resolution in combination with advanced quantum dynamics simulations of the experimental observables using the newly developed QD-DFT/MRCI(2) coupled with the multi-layer multiconfigurational time-dependent Hartree (ML-MCTDH) approach

10. Page 17, lines 364-368: this seems to be guideline or template text. Please check

We thank the referee for pointing this out. This text was inadvertently left from the template/guidelines and should have been removed prior to submission. It has now been deleted.

11. Finally, I suggest to revise the title to reflect more directly the main message of the paper. This is, however, a matter of taste and thus optional and for the authors to decide

The title of the manuscript was deliberately chosen to emphasize the main finding of our study, namely the observation of a previously unreported mechanism occurring during the torsional motion of a fundamental chemical bond: the carbon–carbon double bond. We believe this represents a key aspect of the work and therefore consider the current title to be appropriate.

Reviewer 2:
We thank the referee for the careful reading of our manuscript and for the constructive comments. We appreciate that the referee recognizes that the improved time resolution achieved in our study enables the observation of previously unresolved dynamical features in the TRPES of ethylene.

In particular, our combined experimental and theoretical analysis supports the interpretation that these features arise from the ultrafast population of the dark $\sigma\pi^*$ state following excitation to the

bright $\pi\pi^*$ state.

The referee raises several important questions regarding the modeling and the interpretation, in particular concerning the choice of electronic basis and its role in supporting these conclusions. We address these points in detail below.

1. Following down this line, the choice of diabatic states is not unique and one could have chosen a different diabatic basis, leading to yet another alternative interpretation. From the shape of the adiabatic curves (Figure 1a), one could have, for instance, expected diabatic curves much closer to the adiabatic ones for large torsional angles, and which are energetically degenerate and ≪ cross ≫ around phi=0. Can the authors comment on this alternative choice of diabatic states? It may be that the diabatic states are chosen so that their energy should be equal to the adiabatic ones for a planar geometry, meaning a diabatic energy gap equal to the adiabatic one and a zero coupling at phi=0. This can be a choice but does not necessarily need to be that way. What diabatic states (and subsequent interpretation) would be obtained in case of a choice of a 50/50 mix at phi=0?

We thank the referee for this comment and readily acknowledge that the choice of electronic basis is arbitrary, and furthermore, any quasi-diabatic basis is itself not unique. However, we would argue that this does not mean that each electronic basis is equally *useful* for providing insight into the electronic character of the evolving vibronic wave packet.
The choice of the ground state minimum as the point at which to set the gauge of the adiabatic-to-diabatic transformation is that the $\pi\pi^*$ and $\sigma\pi^*$ are well-defined at this point. Since it is the early time dynamics that are of interest here, choosing an electronic basis that is easily recognizable (in terms of chemical conceptions of electronic states being described by a single dominant electronic configuration) at time $t = 0$ is also particularly convenient in order to track the evolution of this character. Indeed, it is the observation of the rapid change from $\pi\pi^*$ to $\sigma\pi^*$ that is a main contribution of this work.
We believe that while this choice of basis is not unique, it does provide a novel interpretation of the vibronic dynamics of ethylene (a benchmark system), but more importantly, creates an avenue for further inquiry and a route to potential approaches for influencing these dynamics based on, for example, chemical substitution. Specifically, since this study indicates that it is the strong coupling of these two states that gives rise to the rapid, large amplitude torsion, substitution schemes that affect this coupling should also therefore provide a route to potentially controlling this dynamics. It is the practical *utility* of this basis in providing chemical insight that indicates its value. To emphasize that this choice of basis is one that is made in order to provide chemical insight into these dynamics, the following text has been amended in the manuscript:

In contrast to adiabatic states, diabatic states preserve electronic character with respect to nuclear displacements. While the definition of these states is not unique, choosing diabatic states that can be understood in terms of chemical concepts, such as simple orbital occupations, greatly aids in the interpretation of results.

2. Hamiltonian models are often obtained through a least-square fitting procedure against ab initio points, and comparisons of the fitted adiabatic models are typically shown to validate the model with respect to the ab initio points. Here, it is mentioned that the fitting procedure was performed directly in a quasi-diabatic basis. The authors should show the comparison between the fitted model and the quasi-diabatic potential points.

This comparison is now included as Figure S4 in the Supplementary Information. As the figure shows, the fitted diabatic potential curves reproduce the *ab initio* points quantitatively.

3. The out-of-plane twisting normal mode is replaced with a curvilinear torsional coordinate. Have the other normal modes been re-orthogonalised with respect to this torsional coordinate?

The referee is correct to note this approximation is not discussed extensively in the text, as much of this explanation can be found in the Supplementary Information. In short, we assume that

the single a_u-symmetry torsional mode does not couple directly to the other nuclear coordinates and thus no re-orthogonalization is necessary. This follows a similar approximation in Ref. 13 [Schuurman et al., Ann. Rev. Phys. Chem. **69**, 427–450 (2018)] of the main text, which employed a directly analogous nuclear coordinate basis, but a different electronic basis. We assume this approximation is reasonable on the basis of the previous analysis in Ref. 13 of the main text, as well as the accuracy in our simulated absorption spectrum.

To more explicitly acknowledge this approximation in the main body of the manuscript, we have added the following text:

Consistent with previous quantum dynamical studies [54], the a_u-symmetry torsional normal mode coordinate has been replaced with the a curvilinear dihedral angle coordinate in order to better describe large amplitude twisting motion. Details of this approximation can be found in the Supplementary Information.

4. Page 7: "the decrease in vertical ionisation energy with increasing torsional angle". Is it not the increase of ionisation energy?

We thank the referee for pointing this out. It has now been corrected.

Reviewer 3:
We thank the referee for this very positive and thoughtful assessment of our work. We are particularly pleased that the referee recognizes the advantages of the sub-4 fs VUV pulses generated via RDW emission, and the resulting improvement in temporal resolution, which enables the observation of previously unresolved ultrafast dynamics. We also appreciate the referee's acknowledgment of the quality of the experimental data and the level of agreement achieved with the quantum dynamics simulations.

We address below the two points raised by the referee:

1. Population transfer to the ground state. The experimental datasets extend to 120 fs delay; the simulations instead stop at 30 fs. While the limited temporal window is still sufficient to discuss the main point of the paper - the coupling between $\pi\pi^*$ and $\sigma\pi^*$ states - I believe it would be very important to extend the simulations to longer delays to catch the ground state recovery and complete the story. For instance, according to ref. 24, "The nuclear wave packet created by a 160 nm pump pulse accesses the C-C twisted geometries within 10 fs, and the population transfer from the excited to the ground state occurs within the next 20-30 fs"; the authors instead comment on p. 10 that no electronic state other than $\sigma\pi^*$ is significantly populated within the simulation time (and then corroborate this in Figure S3). The rapidly decreasing kinetic energy in the experimental datasets still suggests that the ground state is starting to recover already within the first 100 fs; however, the probe energy limits the observation window and doesn't allow for a direct identification, as it's common for VUV-probe TRPES. Still, given the excellent agreement with theory in the first 30 fs, I believe that the simulations alone would be sufficient in determining the timing of ground state recovery.

We thank the referee for the comment. Unfortunately, a property of the potential expansions in these vibronic Hamiltonians is that they generally will fail to describe relaxation to the ground state via conical intersections. This aspect of the dynamics involves large amplitude nuclear displacements along multiple coordinates. As the potential expansion here is a Taylor-expansion in all coordinates but the torsional mode, the potential will fail to describe even qualitatively the known relaxation pathways that involve coupled twist-pyramidalization and/or H-transfer processes. For this reason, we limit the analysis and discussion to the short-time dynamics that give rise to the initial torsional motion in the wave packet.

We can nevertheless compare our experimental results with the study performed by the group of T. Suzuki using an extreme-ultraviolet probe pulse [2]. As mention by the referee, the authors concluded in this work that, following excitation at a pump wavelength of 160 nm, the nuclear wavepacket reaches the CC twisted geometry within approximately 10 fs, while population transfer

from the excited to the ground state occurs over the subsequent 20–30 fs.

Our experimental investigation supports this interpretation, while providing improved temporal resolution. We likewise observe that the nuclear wavepacket reaches the CC twisted geometry within 10±1 fs, and we measure a decay of the photoelectron signal on a timescale of 25.9±0.5 fs, in excellent agreement with the previous study.

However, because our observable is not directly sensitive to the ground-state population, owing to the limited photon energy of the probe laser pulse, the decay observed in the TRPES cannot be unambiguously assigned to ground-state recovery. Additional measurement, combining for instance ultrashort VUV pulses obtained by RDW emission and attosecond XUV pulses obtained by HHG will be required.

2. Identification of $\pi\pi^*$ and $\sigma\pi^*$ states via PADs. As the experiment is performed with a VMI, I believe it would be of interest to determine whether the available angular resolution can play a role in aiding the interpretation. Could the authors comment on the capability to distinguish $\pi\pi^*$ - and $\sigma\pi^*$ -originated photoelectrons based on their angular distributions (PADs)?

We thank the referee for this insightful suggestion. We agree that photoelectron angular distributions (PADs) can, in principle, provide valuable information for distinguishing ionization originating from the $\pi\pi^*$ and $\sigma\pi^*$ excited states.

To address this point, we analyzed the PADs extracted from the VMI measurements and evaluated the corresponding anisotropy parameters, β_2 and β_4, which characterize the angular distribution of photoelectrons generated in the two-photon ionization process. Small variations in these parameters are indeed observed (see Fig. 1(a) and (b) below). Nevertheless, the observed differences remain relatively subtle and do not allow for an unambiguous assignment of the photoelectron signal to either the $\pi\pi^*$ or $\sigma\pi^*$ state.

This behavior can be rationalized using symmetry considerations. For ethylene near the Franck–Condon geometry, where the molecular symmetry is described by D_{2h}, the symmetry-allowed ionization transition must satisfy

$$\Gamma^{\text{excited}} \otimes \Gamma^{\mu} \otimes \Gamma^{\text{cation}} \otimes \Gamma^{\text{free electron}} = A_{1g}, \tag{1}$$

where Γ^{excited}, Γ^{μ}, Γ^{cation}, and $\Gamma^{\text{free electron}}$ denote the irreducible representations of the excited electronic state, dipole operator, ionic state, and outgoing photoelectron continuum, respectively.

The $\pi\pi^*$ state of ethylene possesses B_{2u} symmetry at the Franck–Condon geometry (see Supporting Information, section "Analysis of the ground-state VUV absorption spectrum" for the assigned symmetries of the neutral and ionic states at the Franck–Condon point), whereas the $\sigma\pi^*$ state has B_{2g} symmetry. At the Franck-Condon point, the photoelectron partial wave decomposition for the $|\pi\pi^*\rangle \rightarrow |\pi^1\rangle$ ionization channel is expected to contain p and f angular momentum whereas for $|\sigma\pi^*\rangle \rightarrow |\pi^1\rangle$ ionization channel, it is expected to contain s and d partial wave. This is in agreement with our experimental observation. At the zero delay, the photoelectron associated with the $|\pi\pi^*\rangle \rightarrow |\pi^1\rangle$ ionization channel peaks along the laser polarization (β_2 ∼1.4) as expected for a $p-$wave like electronic wavefunction. At a later time, around ∼22 fs, the photoelectron signal observed at ∼4.3 eV is assigned to the $|\sigma\pi^*\rangle \rightarrow |\pi^1\rangle$ ionization channel following the return of the vibronic wavepacket to the Franck-Condon region. In this case, the measured anisotropy parameter decreases to $\beta_2 \sim 0.8$, substantially smaller than the value observed at zero delay, where ionization from the $|\pi\pi^*\rangle$ state dominates. This reduction is likewise consistent with the expected s- and d-wave character of the $|\sigma\pi^*\rangle \rightarrow |\pi^1\rangle$ ionization channel. We therefore agree with the referee that, near the Franck–Condon geometry, one would expect significantly different partial-wave characters for the emitted photoelectron.

However, following excitation to the $\pi\pi^*$ state, ethylene undergoes substantial torsional motion, which drives the molecule away from the equilibrium D_{2h} geometry. As the torsional angle approaches 90°, the molecular symmetry evolves toward D_{2d} through intermediate D_2 geometries, reducing the applicability of strict D_{2h} selection rules. In D_2 symmetry, due to the loss of mirror-

plane symmetry and inversion symmetry, both the $\pi\pi^*$ and $\sigma\pi^*$ states correlate to the same B_2 irreducible representation and therefore become symmetry-indistinguishable. Consequently, outside of the equilibrium geometry, it is no longer possible to distinguish whether the ionization is taking place from the $\pi\pi^*$ or $\sigma\pi^*$ state solely based on their PADs. In conclusion, an unambiguous assignment of the photoelectron signal to either electronic state using PADs alone is not straightforward.

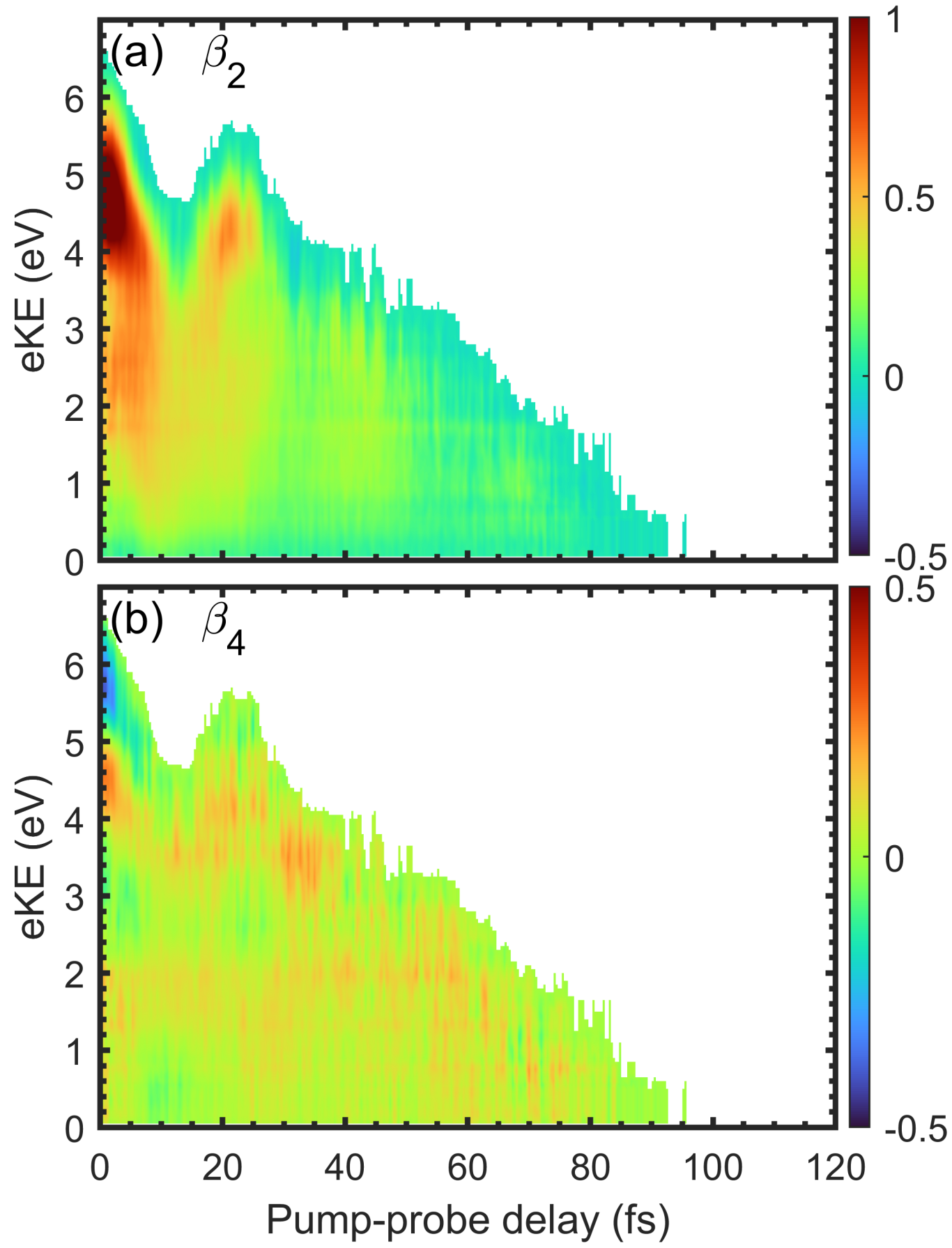

Fig. 1: Time- and energy-resolved photoelectron angular anisotropy parameters of C_2H_4: (a) $\beta_2(E_k, t)$ and (b) $\beta_4(E_k, t)$.

*References

[1] A. Boyer, A. Humeniuk, S. Karashima, and T. Suzuki. Deuterium isotope effect on internal conversion of ethylene studied by time-resolved photoelectron spectroscopy. *J. Phys. Chem. A*, 128:7068–7072, 2024.

[2] S. Karashima, A. Humeniuk, W. J. Glover, and T. Suzuki. Ultrafast photoisomerization of ethylene studied using time-resolved Extreme Ultraviolet photoelectron spectroscopy. *J. Phys. Chem. A*, 126:3873–3879, 2022.

We thank all three reviewers for their careful evaluation of our manuscript and for their constructive comments throughout the review process. We have addressed the remaining comments raised during the second round of review and revised the manuscript accordingly. Our detailed responses to each of these comments are provided in the point-by-point reply below.

Reviewer 1:

We sincerely thank the reviewer for the careful evaluation of our manuscript and for recommending its publication. We appreciate the positive assessment and have no further response.

Reviewer 2:

We thank the referee for the careful reading of our revised manuscript and for the constructive comments. The referee raises additional question regarding the modeling and the interpretation. We address this point in detail below.

1. The authors have addressed my comments. They have in particular included in the SI (Figure S4) the comparison of the Hamiltonian model against the ab initio electronic structure data. I note a discrepancy along normal mode 8, with the fitted model going down in energy for large positive Q values. Can the authors comment on this discrepancy and the potential impact it may have on the dynamics?

This particular state corresponds to a Rydberg state that is not appreciably populated during the dynamics, and thus, the influence on the results is minimal. The "turnover" of this potential curve could be corrected with additional data at large displacements, but since the state does not participate in the dynamics following excitation to either the $\pi\pi^*$ or $\sigma\pi^*$, and an artifactual maximum occurs at energies above the pump energy, this was not required for the current study.

Reviewer 3:

We thank the reviewer for this very positive and thoughtful assessment of our work. We are particularly grateful for the reviewer's recommendation that the manuscript is suitable for publication. We also appreciate the additional constructive suggestions, which have further improved the clarity and completeness of the manuscript.

We address the two points raised by the reviewer below.

1. It's indeed a pity that the vibronic Hamiltonian fails to describe relaxation to the ground state: I now fully understand the point. I thus suggest the authors to point this out in the main text, as the limitations of the employed model may not be straightforward to the non-specialized reader. For the same reason, I also suggest to mention the experimental limitations related to the observation window in following the relaxation pathway back to the ground state.

We thank the reviewer for this valuable suggestion. We agree that explicitly stating both the theoretical and experimental limitations will improve the clarity of the manuscript, particularly for non-specialist readers. To reflect this in the manuscript, we have added the following text in the discussion:

The potential was represented by a Taylor expansion in all coordinates except the torsional mode. As a result, the resulting model could not describe, even qualitatively, the known relaxation pathways involving coupled twist-pyramidalization and/or H-transfer processes. For this reason, we

limit the analysis and discussion to the short-time dynamics that give rise to the initial torsional motion in the wave packet.

2. I appreciate the analysis of the asymmetry parameters and the related discussion provided by the authors. I suggest the authors to include it in the Supplementary Informations, as it's a very useful addition for the TRPES community.

We thank the reviewer for this positive suggestion. We agree that the photoelectron angular distribution analysis may be valuable to readers working in time-resolved photoelectron spectroscopy. Accordingly, we have included the complete asymmetry-parameter analysis and the associated discussion in the Supplementary Information, together with the corresponding figures and explanatory text, as suggested.
